# A Multidisciplinary Team–Based Large Language Model Framework for Predicting Postoperative Neurological Complications in Acute Type A Aortic Dissection: Model Development and Validation Study

**DOI:** 10.2196/91257

**Published:** 2026-08-18

**Authors:** Jili Li, Julin Zhang, Xingrui Tao, Yaoye Chen, Siqi Xun, Guangshuo Qin, Kaiyue Diao, Chaoyi Qin

**Affiliations:** 1 Department of Thoracic Surgery and Institute of Thoracic Oncology, West China Hospital, Sichuan University, Chengdu, Sichuan, China; 2 Department of Cardiovascular Surgery and Cardiovascular Surgery Research Laboratory, West China Hospital, Sichuan University, No. 37, Guoxue Alley, Chengdu, Sichuan, 610041, China, 86 028 85422897; 3 West China School of Medicine, West China Hospital, Sichuan University, Chengdu, Sichuan, China; 4 School of Computer Science, Shanghai Jiao Tong University, Shanghai, China; 5 Department of Radiology, West China Hospital, Sichuan University, Chengdu, Sichuan, China

**Keywords:** acute type A aortic dissection, postoperative neurological complications, large language model, multidisciplinary team, in-context learning, machine learning

## Abstract

**Background:**

Postoperative neurological complications (PNCs) after acute type A aortic dissection (ATAAD) surgery are clinically emergent and require multidimensional perioperative risk assessment. Large language models (LLMs) have shown potential in clinical prediction, but the incremental value of structured multiagent collaboration remains unclear.

**Objective:**

This study aimed to develop and validate a multidisciplinary team (MDT)–based LLM framework for predicting PNC after ATAAD surgery and to compare its performance with that of traditional machine learning (ML) models and single-agent LLM settings.

**Methods:**

A retrospective cohort from January 2020 to June 2024 (N=763) was randomly divided into a training set (n=533) and an internal validation set (n=230). A prospective cohort from July 2024 to June 2025 (n=120) was used for prospective validation. The outcome was PNC, defined as stroke, cerebral hemorrhage, paraplegia, or coma. Population-level in-context learning used outcome-stratified summary statistics from the training cohort, including predictor distributions in patients with and without PNC and between-group *P* values. Two LLMs (DeepSeek-V3 and ChatGPT [GPT-5]) were evaluated under 4 settings: no MDT without in-context learning, MDT without in-context learning, no MDT with in-context learning, and MDT with in-context learning. Model performance was assessed using the area under the receiver operating characteristic curve (AUC), sensitivity, specificity, accuracy, *F*_1_-score, and Brier score.

**Results:**

PNC occurred in 13.0% (99/763) of patients in the retrospective cohort and 15.8% (19/120) in the prospective cohort. Among the ML models, the random forest achieved the highest AUC in the internal validation set (AUC 0.7857, 95% CI 0.6945‐0.8768). Among the LLM configurations, GPT-5 with MDT and in-context learning achieved the highest AUC (AUC 0.8419, 95% CI 0.7398‐0.9440), with a sensitivity of 80.00%, specificity of 87.00%, and Brier score of 0.0876. Although this configuration showed a significantly higher AUC than the fully unaided GPT-5 baseline (*P*=.006), this improvement reflected the combined effect of MDT and in-context learning, as adding the MDT framework within matched settings did not significantly improve AUC over corresponding single-agent settings in either cohort. The AUC of GPT-5 with MDT and in-context learning was also not significantly higher than that of the random forest model (*P*=.18). Word frequency analysis showed that the generated rationales following in-context learning more frequently mentioned variables that were statistically significant in the provided context.

**Conclusions:**

The GPT-5 configuration combining MDT-style collaboration with population-level in-context learning showed promising discrimination for PNC prediction after ATAAD surgery. However, the MDT layer did not produce a statistically significant incremental improvement in AUC over the corresponding single-agent settings, and its predictive contribution remains to be confirmed. The framework generated structured, role-specific rationales, supporting its further evaluation as a proof-of-concept approach for postoperative risk stratification. Larger multicenter studies are required before routine clinical implementation.

## Introduction

Prediction plays a critical role in the health care domain, with common application scenarios including assessing disease mortality [1-3], evaluating drug suitability [4-6], and determining patient discharge readiness [7,8]. Traditional machine learning (ML) methods have been widely used in clinical prediction tasks and have demonstrated good performance [9,10]. With the advancement of large language models (LLMs), various models, such as ChatGPT and the DeepSeek series, have emerged. These models perform well in text understanding and generation [11,12], and their application in clinical prediction tasks is increasing [13,14]. Prior studies have also evaluated LLMs using standardized clinical vignettes [15], physician-authored case summaries [16], or structured electronic health record information converted into narrative prompts [17,18]. However, because LLMs are built upon diverse, general-purpose databases not specifically designed for the clinical domain, they possess universality and broad-spectrum applicability that can limit their professional use in clinical applications. A study indicated that LLMs perform less effectively than locally trained traditional ML models in clinical prediction tasks [19] and that they exhibit limitations when handling complex cases [20]. Furthermore, the real-world “out-of-the-box” performance of LLMs in actual health care settings remains unclear, and strategies for enhancing their capabilities through precise in-context learning require further exploration.

Acute type A aortic dissection (ATAAD) is a life-threatening cardiovascular emergency [21,22]. Surgical intervention remains the primary treatment for patients with ATAAD. While surgical mortality for ATAAD has significantly decreased with advancements in surgical techniques, postoperative in-hospital mortality remains relatively high, reported to be up to 20% [23]. The occurrence of postoperative neurological complications (PNCs) significantly impacts patient outcomes, leading to postoperative cognitive dysfunction and an increased risk of in-hospital mortality. Therefore, the timely prediction, identification, and intervention for PNC represent a crucial and valuable clinical task for improving patient outcomes. The probability of PNC is influenced by multiple preoperative and intraoperative factors, such as preoperative altered mental status [24], intraoperative peak lactate level [25], cannulation site [26], and cerebral perfusion strategies [27]. This typically necessitates collaborative diagnosis and decision-making by a multidisciplinary medical team comprising specialists in cardiac surgery, anesthesiology, and neurology.

Currently, manually organized multidisciplinary team (MDT) medical consultations consume substantial human, material, and financial resources [28]. Moreover, ATAAD presents abruptly and progresses rapidly. Without timely intervention, the mortality rate reaches as high as 50% within 48 hours, increasing by 1% to 2% per hour after symptom onset. Given the critical time constraints for patients, there is often insufficient time for conventional MDT medical consultations, thus creating an urgent need for a more convenient and efficient collaborative diagnostic approach. In clinical applications, different agents can be designed to organize structured, role-specific discussions, inspired by MDT collaboration, for complex medical issues [29,30], although such systems are not equivalent to real MDT consultations that also involve bedside examination, imaging review, longitudinal patient knowledge, and nuanced clinical judgment [31]. The multiagent framework represents an innovative approach that significantly enhances the capabilities of LLMs to handle complex tasks [32,33] by enabling multiple agents to engage in discussions around the same problem and ultimately reach a consensus on the output. Simultaneously, the resulting conversation provides a traceable record of role-specific reasoning, allowing health care professionals to review the model’s deliberation pathway and increasing its credibility in clinical applications.

This study aims to develop an MDT framework for clinical prediction tasks. By using an in-context learning method, LLMs can learn from typical clinical features and reduce the limitations imposed by the length of the context text on the number of training cases. In particular, we systematically compare the predictive performance of traditional ML, single agent–based LLMs, and MDT framework–based LLMs in assessing the risk of PNC following ATAAD surgery.

## Methods

### Ethical Considerations

This study was conducted in accordance with the Declaration of Helsinki and was approved by the Institutional Review Board of West China Hospital, Sichuan University (2023-1999). Given the retrospective nature of the initial cohort development and the anonymization of data, the requirement for informed consent was waived. However, written informed consent was obtained from all participants enrolled in the prospective validation cohort. To ensure privacy and confidentiality, all patient data were deidentified prior to analysis. In accordance with our data use agreement, all prompts transmitted to the LLM application programming interfaces were rigorously monitored to ensure that no protected health information was included.

### Study Population

This study consisted of 2 independent, nonoverlapping cohorts of patients diagnosed with ATAAD at West China Hospital, Sichuan University. In the retrospective cohort derived from the electronic medical record system, after excluding those with chronic aortic dissection (n=139) from an initial pool of 902 patients, a total of 763 patients diagnosed with ATAAD were included. The retrospective cohort (January 2020 to June 2024, n=763) was used for model development and internal validation, while the prospective cohort (July 2024 to June 2025, n=120) was used for prospective validation. The predicted outcome was the occurrence of PNC, defined as a composite of stroke, cerebral hemorrhage, paraplegia, or coma. Stroke was defined as a new focal or global neurological deficit attributable to cerebral ischemia, with compatible cranial computed tomography or magnetic resonance imaging findings when imaging was available or clinically indicated. Cerebral hemorrhage was defined as postoperative intracranial hemorrhage confirmed by cranial computed tomography or magnetic resonance imaging. Paraplegia was defined as a new postoperative bilateral lower-extremity motor deficit consistent with spinal cord ischemia and documented by neurological examination during hospitalization. Coma was defined as unarousable unconsciousness without purposeful response to verbal or painful stimulation lasting more than 48 hours after surgery after excluding residual anesthesia or sedation. Predictors encompassed demographics, comorbidities, laboratory profiles, imaging findings, and operative details. The intended clinical use of this framework was postoperative risk assessment and early prognostic stratification for PNC after ATAAD surgery. Therefore, the predictor set was defined to include complete perioperative information, including intraoperative variables, to reflect the way postoperative neurological risk is commonly assessed in routine clinical practice.

The development, analysis, and reporting of this clinical prediction model study were performed in accordance with the TRIPOD+AI (Transparent Reporting of a Multivariable Prediction Model for Individual Prognosis or Diagnosis + AI) statement (Checklist 1), which provides updated guidance for reporting prediction models developed using regression or machine learning methods [34]. For LLM-based prediction, the prespecified variables extracted from the electronic medical records were further transformed into standardized clinical vignettes. A uniform vignette template was manually designed based on the study variables, and Python scripts were used to batch-convert the structured variables into narrative case descriptions for model input. This process was completed by 1 author (SX), who had access only to the predictor variables and was blinded to the prediction outcomes, thereby reducing the risk of information bias.

### ML Models and Explainability

Four machine learning models (random forest, extreme gradient boosting, Gaussian Naive Bayes, and logistic regression) were developed to predict PNC. For the primary analysis, the ML models were trained and evaluated using the same set of predictor variables as those provided to the LLMs, including demographics, comorbidities, laboratory profiles, imaging findings, and operative variables. As a sensitivity analysis, we additionally repeated the ML modeling using the 15 predictors selected by least absolute shrinkage and selection operator (LASSO) regression, which was performed strictly within the training set of 533 patients to reduce model dimensionality and mitigate overfitting. LASSO regression was performed using the glmnet R package (version 4.1-10; R Foundation for Statistical Computing), with categorical predictors one-hot encoded and predictors standardized before model fitting. The penalty parameter λ was selected by 5-fold cross-validation using the value that minimized cross-validated binomial deviance. Predictors with nonzero coefficients at the optimal λ were retained for the sensitivity analysis. The corresponding results are presented in Multimedia Appendix 1. The random forest model, which demonstrated the highest predictive performance among ML models, was subsequently analyzed to quantify the global importance of each input predictor using Shapley Additive Explanations (SHAP) [35].

### LLM In-Context Learning

DeepSeek-V3 and ChatGPT (GPT-5), which are increasingly being evaluated for clinical applications, were selected as the base models. The temperature for DeepSeek-V3 was set to 0 to reduce output stochasticity and improve reproducibility. For GPT-5, which does not support a user-controlled temperature parameter according to the official document, we used the default model configuration. To assess the reproducibility of GPT-5 predictions under the default model configuration, we conducted a repeated-inference analysis on a stratified random sample of 50 patients from the retrospective cohort, selected according to the observed outcome distribution. For each patient, inference was independently repeated 5 times under identical prompting conditions across all 4 GPT-5 application settings, using all clinical variables. Within-patient variability of predicted probabilities was quantified using the SD, range, and IQR.

In addition, we adopted an in-context learning strategy to prime the LLMs using aggregated, population-level summary statistics. Rather than updating model parameters or fine-tuning the models, these statistics were injected into the system prompt at inference time as structured contextual information. This approach avoided the computational cost of fine-tuning while providing the models with essential background knowledge. The background knowledge, formatted in JSON, included distributions of the predictors across the entire training cohort, the PNC group, and the non-PNC group, along with *P* values from intergroup comparisons (Multimedia Appendix 1). This summary enabled the LLMs to recognize key risk factors and population characteristics associated with PNC. By leveraging these statistical summaries, the LLMs established a foundational understanding of the clinical dataset, supporting subsequent reasoning for individual patient predictions and ensuring an equitable comparison with traditional ML models.

For the primary analysis, both the cohort-level background knowledge and the patient-specific prompts were constructed using exactly the same full set of structured clinical variables available in the original dataset, as that used for the ML models, thereby ensuring a fair comparison. As a sensitivity analysis, the LLM-based predictions were repeated using the same 15 predictors selected by LASSO regression, and the background knowledge also contained only the selected variables (Multimedia Appendix 1).

### Multiagent Collaborative Framework

**Figure 1.**
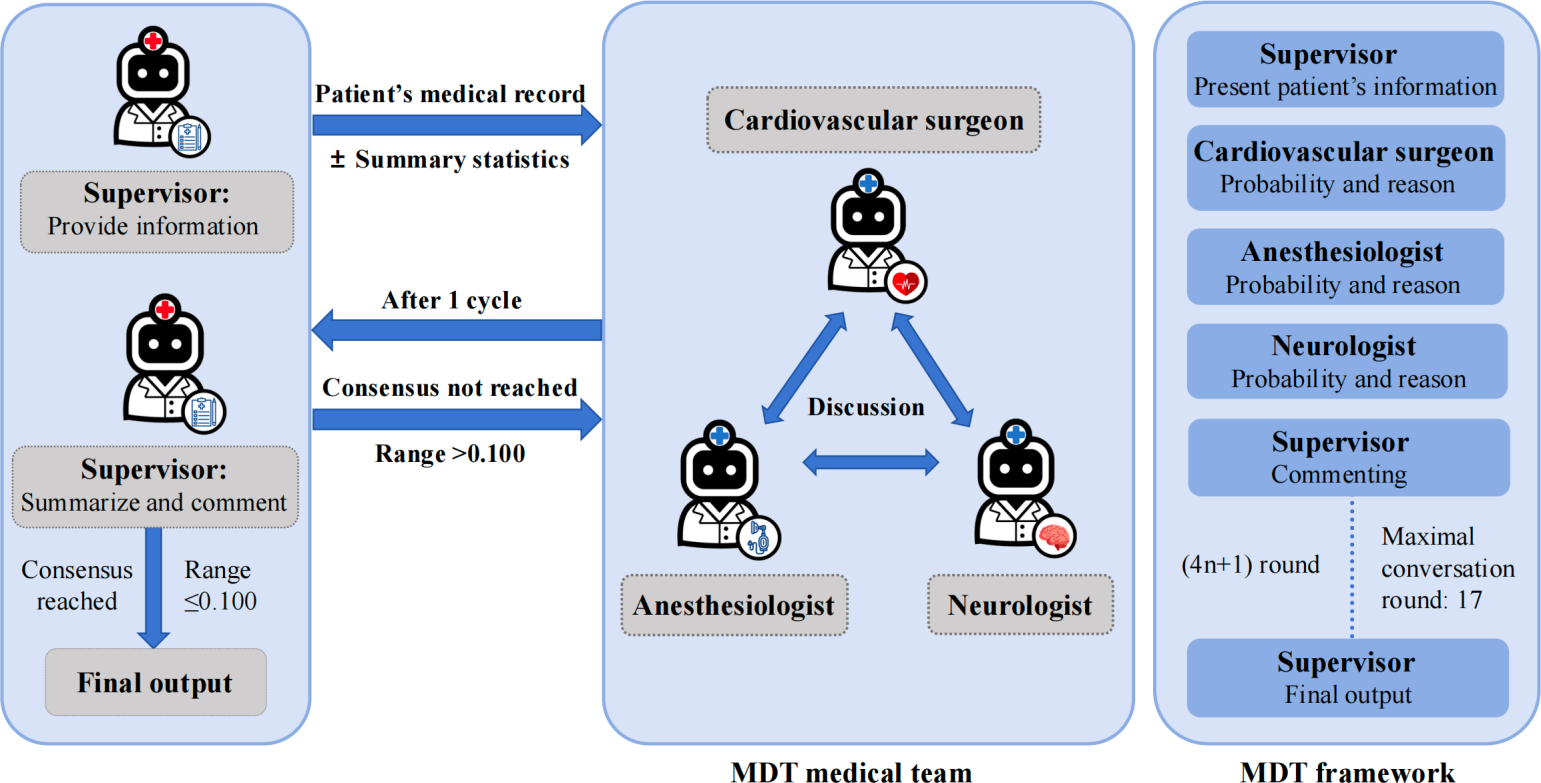
Schematic of the multiagent collaborative framework for predicting postoperative neurological complications. The multidisciplinary team (MDT) framework, built with AutoGen, comprises 3 specialist agents (cardiovascular surgeon, anesthesiologist, and neurologist) and a supervisor agent, simulating an MDT consultation to reach a consensus-based prediction.

The same models were selected for our multiagent conversation framework developed using AutoGen (Microsoft) [36]. As shown in Figure 1, this framework simulates MDT consultations for predicting PNC. The framework comprises 3 specialized agents (cardiovascular surgeon, anesthesiologist, and neurologist) and 1 supervisor agent. Each specialist agent analyzes the case from a distinct perspective: the cardiovascular surgeon focuses on surgical factors and anatomy, the anesthesiologist evaluates intraoperative management and hemodynamics, and the neurologist assesses neurological status and cerebrovascular conditions. For the no MDT baseline, predictions were generated by the single cardiovascular surgeon agent because patients undergoing ATAAD surgery are primarily managed in the cardiovascular surgery ward after surgery, and early postoperative risk assessment is commonly initiated by cardiovascular surgeons in routine clinical practice. The same single-agent role and prompt schema were used for both DeepSeek-V3 and GPT-5 under the no MDT without in-context learning and no MDT with in-context learning settings. The complete prompts for these 2 no MDT settings are provided in Multimedia Appendix 1.

For each patient case, the conversation was initialized with an empty message history, so no conversational state was carried over between cases. Patient-specific narrative prompts were generated from the completed dataset after imputation, and imputed values were not explicitly labeled or distinguished from observed clinical measurements in the prompts. Within each case-level session, the dialog state was maintained through the accumulated group-chat history. The dialog followed a structured framework, initiated by the supervisor, who presented the patient’s information and guided each specialist agent to provide a unique probability and rationale. After each discussion cycle, the supervisor coordinated exchanges and monitored for consensus, defined as a probability range of ≤0.1. Once consensus was reached, or after a maximum of 17 rounds, the dialog terminated. The supervisor then produced the average probability and a summary rationale, integrating complementary expertise through a structured MDT-style deliberation. The complete conversation history and the final supervisor summary were saved for subsequent analysis. To characterize the content emphasized in the multiagent outputs, we conducted a word-frequency analysis of the summary rationales generated by the supervisor.

### Statistical Analysis

We handled missing data using multiple imputation by chained equations (MICE), a well-established approach for addressing uncertainty in imputed values [37]. For the retrospective cohort, missing continuous predictors were imputed using MICE with 5 imputed datasets and 10 iterations, and the averaged values were then used to construct the completed dataset. For the prospective validation cohort, we enrolled 120 consecutive eligible patients for whom the prespecified predictor variables were fully available, and therefore no imputation was performed. In addition, to ensure a fair comparison, traditional ML models and LLM-based models were evaluated using the same set of predictor variables on the completed dataset after imputation. Continuous variables were assessed for normality using the Shapiro-Wilk test. Nonnormally distributed variables were summarized as medians with IQRs and compared using the Mann-Whitney *U* test, whereas normally distributed variables were presented as means with SDs and compared using the 2-tailed *t* test. Categorical variables were expressed as numbers and proportions, with group comparisons performed using the chi-square test or Fisher exact test, as appropriate.

The performance of both ML models and LLMs was evaluated using the area under the receiver operating characteristic curve (AUC), sensitivity, specificity, positive predictive value (PPV), negative predictive value (NPV), accuracy, *F*_1_-score, and Brier score. To achieve optimal classification performance, the prediction threshold for each model was selected by maximizing the Youden index. The 95% CIs for AUCs were calculated using the DeLong method, whereas those for sensitivity, specificity, PPV, NPV, accuracy, *F*_1_-score, and Brier score were estimated using 1000-iteration nonparametric bootstrap resampling, with the Youden index threshold reestimated in each bootstrap sample for threshold-dependent metrics. To compare discriminative performance between models, differences in AUC were assessed using the DeLong test. All *P* values from model-comparison tests were reported as unadjusted exploratory results, and the study was not powered for confirmatory multimodel hypothesis testing across all LLM configurations, cohorts, and model classes. Decision curve analysis and calibration plots were performed to further assess the clinical utility and probability calibration of the LLM models. All statistical analyses were performed using Python (version 3.13.7; Python Software Foundation) and R software (version 4.5.0).

## Results

### Patient Characteristics

**Figure 2.**
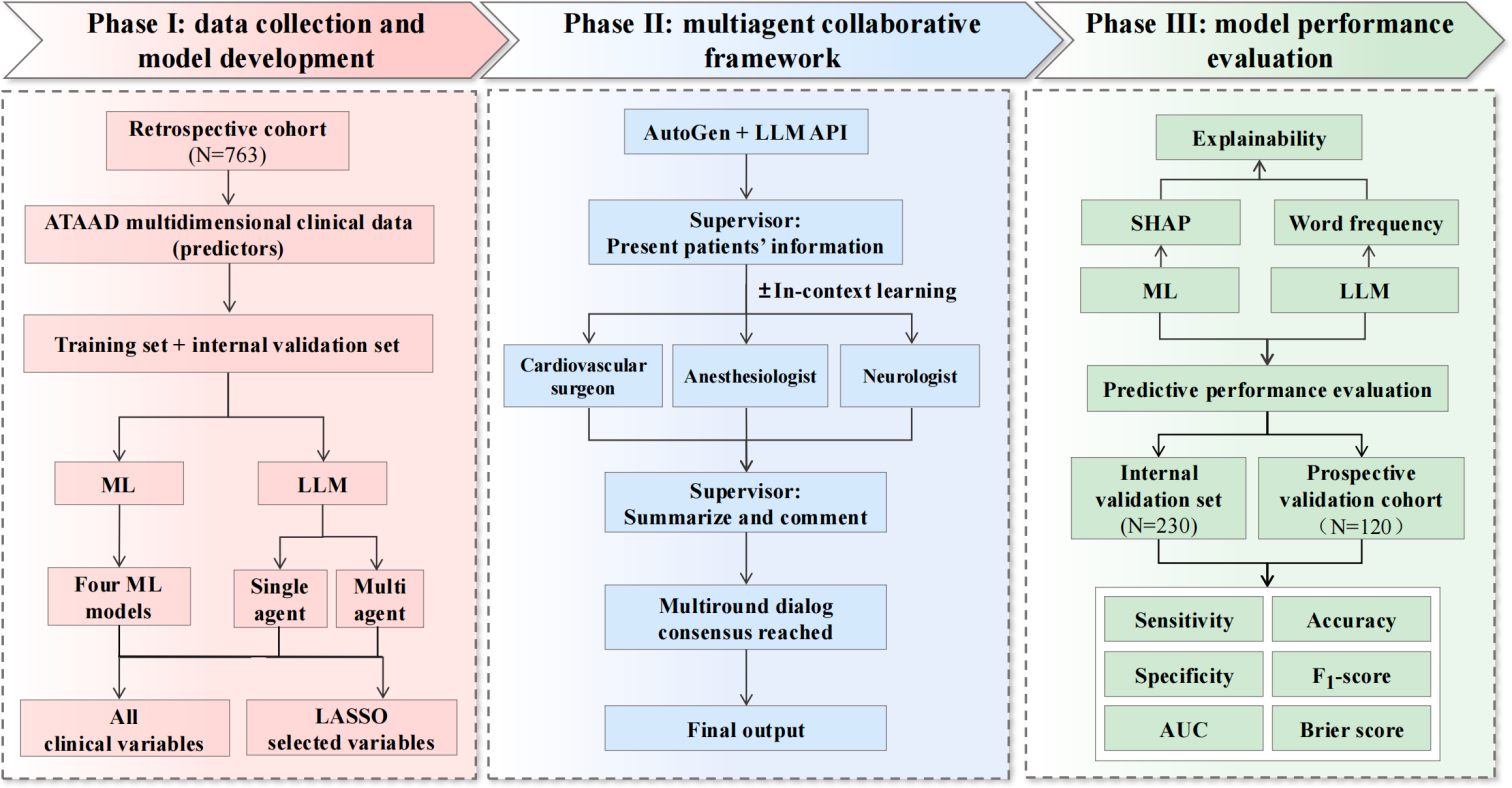
Study workflow for developing and validating machine learning (ML) and large language model (LLM) methods to predict postoperative neurological complications (PNC). The retrospective cohort was randomly divided into a training set and an internal validation set (7:3). After data preprocessing and variable selection, 4 ML models and single-agent or multiagent LLM configurations, with or without population-level in-context learning, were evaluated. Model performance was assessed using AUC, sensitivity, specificity, accuracy, *F*_1_-score, and Brier score, and interpretability was examined using the Shapley Additive Explanations (SHAP) algorithm and word frequency analysis. ATAAD: acute type A aortic dissection; AUC: area under the receiver operating characteristic curve; LASSO: least absolute shrinkage and selection operator.

The study comprised a retrospective cohort of 763 patients and a prospective validation cohort of 120 patients with ATAAD. We randomly allocated the retrospective cohort to a training set (n=533, 70%) and an internal validation set (n=230, 30%). The overall incidence of PNC in the retrospective cohort was 13.0% (99/763). Detailed comparisons of demographic, clinical, and operative characteristics between patients with and without PNC in the retrospective cohort are provided in Table S1 in Multimedia Appendix 1. In the prospective validation cohort, PNC occurred in 19 of 120 (15.8%) patients. The baseline characteristics of the retrospective and prospective validation cohorts are summarized in Table S2 in Multimedia Appendix 1. The details of the study design are presented in Figure 2.

### ML Model and Interpretation

**Figure 3.**
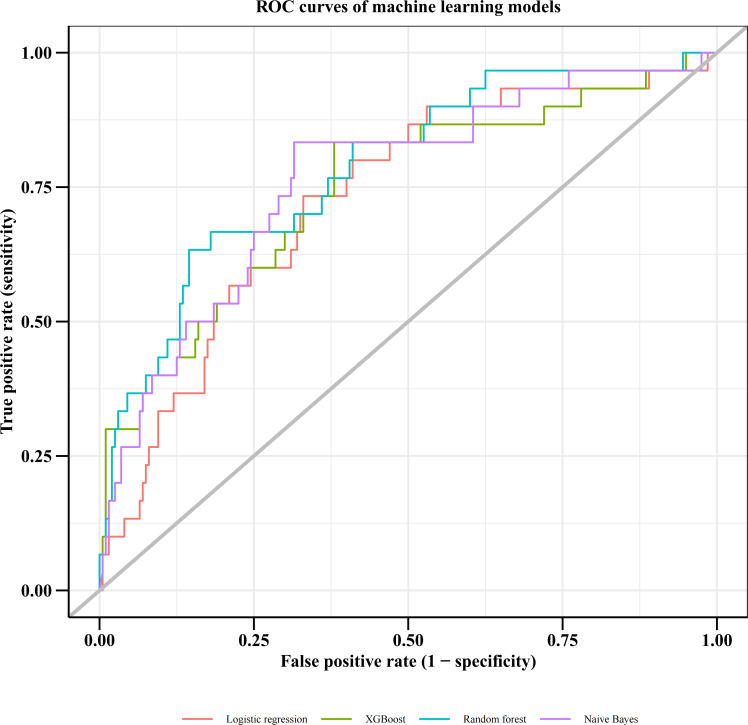
Receiver operating characteristic (ROC) curves of the 4 machine learning models.

We developed 4 ML models using all prespecified candidate predictors, exactly matching the variables provided to the LLMs in the primary analysis. As shown in Table 1 and Figure 3, the random forest achieved the highest AUC on the internal validation set among the 4 ML models (AUC 0.7857, 95% CI 0.6945‐0.8768), whereas logistic regression had the poorest predictive performance (AUC 0.7292, 95% CI 0.6330‐0.8254).

**Table 1.** Performance of each machine learning model for prediction on the internal validation set using all predictor variables.

Model	AUC^a^ (95% CI)	Sensitivity (95% CI)	Specificity (95% CI)	PPV^b^ (95% CI)	NPV^c^ (95% CI)	Accuracy (95% CI)	*F*_1_-score (95% CI)	Brier score (95% CI)
Random Forest	0.7857 (0.6945-0.8768)	0.6333 (0.5217-0.9677)	0.8550 (0.4368-0.9114)	0.3958 (0.1806-0.5557)	0.9396 (0.9124-0.9916)	0.8261 (0.4955-0.8784)	0.4872 (0.3043-0.6119)	0.1020 (0.0727-0.1314)
XGBoost	0.7450 (0.6419-0.8481)	0.8333 (0.4705-0.9565)	0.6200 (0.5604-0.9415)	0.2475 (0.1731-0.6088)	0.9612 (0.9179-0.9919)	0.6478 (0.5957-0.8871)	0.3817 (0.2836-0.5582)	0.1016 (0.0717-0.1328)
Naive Bayes	0.7634 (0.6678-0.8591)	0.8333 (0.6295-0.9600)	0.6850 (0.6231-0.8707)	0.2841 (0.1932-0.4194)	0.9648 (0.9296-0.9926)	0.7043 (0.6478-0.8435)	0.4237 (0.3106-0.5393)	0.2761 (0.2288-0.3313)
Logistic regression	0.7292 (0.6330-0.8254)	0.7333 (0.5454-0.9714)	0.6700 (0.4349-0.8443)	0.2500 (0.1648-0.3969)	0.9437 (0.9130-0.9921)	0.6783 (0.4957-0.8174)	0.3729 (0.2745-0.5037)	0.1141 (0.0839-0.1446)

a AUC: area under the receiver operating characteristic curve.

b PPV: positive predictive value.

c NPV: negative predictive value.

**Figure 4.**
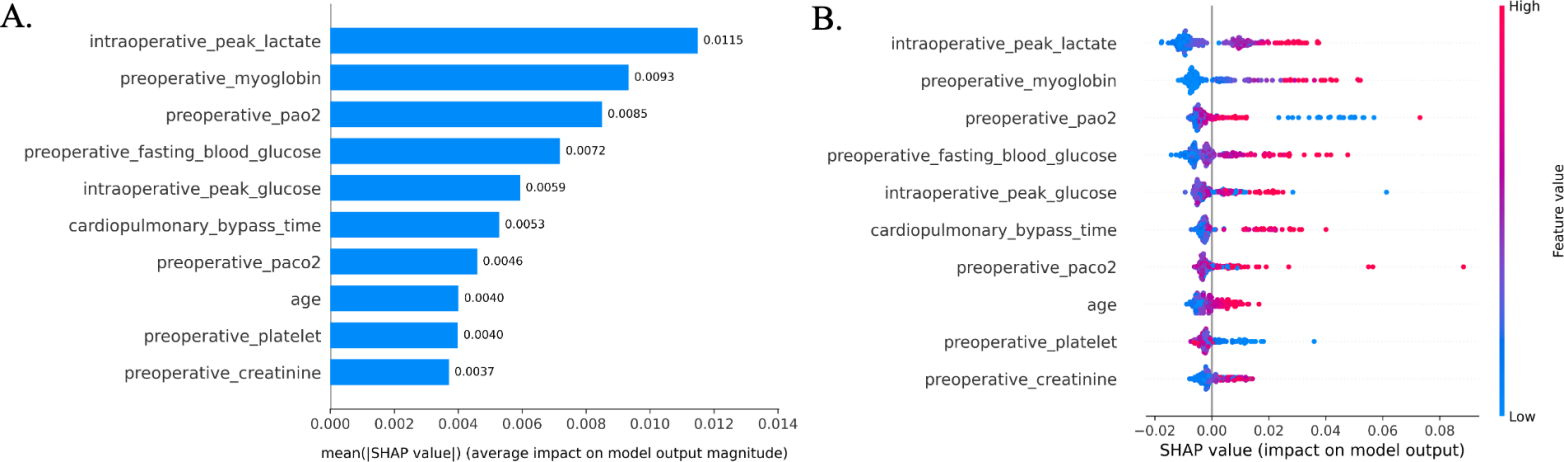
Interpretation of the random forest model using the Shapley Additive Explanations (SHAP) method. (A) Global feature importance is ranked by mean absolute SHAP values. (B) The SHAP summary plot shows the direction and magnitude of each feature’s contribution to the predicted risk of postoperative neurological complications.

To provide visual interpretations of the prediction outcome using the internal validation set, we applied the SHAP algorithm to explain the random forest model and displayed the top 10 predictors ranked by importance in Figure 4A. The intraoperative peak lactate was the most important feature for predicting postoperative PNC, followed closely by the preoperative myoglobin level. The association between the SHAP values of each feature and their effects on the model prediction is presented in Figure 4B. The color of the dots indicates whether the feature value is high (red) or low (blue) for each observation.

### LLM Prediction and Multiagent Evaluation

Repeated inference demonstrated limited within-patient variability across all 4 GPT-5 application settings (Figure S1 in Multimedia Appendix 1). The mean within-patient SD ranged from 0.015 to 0.023, the mean range from 0.036 to 0.054, and the mean IQR from 0.018 to 0.028 (Table S3 in Multimedia Appendix 1). The GPT-5+MDT+context setting showed relatively low variability, with a mean SD of 0.015 and a mean range of 0.036, supporting acceptable empirical reproducibility under fixed prompting conditions. Based on the reproducibility of LLMs, we evaluated the predictive performance of DeepSeek-V3 and GPT-5 under 4 application settings in the internal validation set. As summarized in Table 2, configurations incorporating in-context learning had higher observed AUCs than the corresponding settings without in-context learning. In the internal validation set, GPT-5 achieved a higher AUC than DeepSeek-V3 in each matched configuration. The highest AUC was observed for GPT-5 with both MDT and in-context learning (AUC 0.8419, 95% CI 0.7398‐0.9440), with a sensitivity of 80.00%, specificity of 87.00%, accuracy of 86.09%, and *F*_1_-score of 0.60. Compared with the fully unaided baseline without MDT or in-context learning, the MDT+context configuration showed a significantly higher AUC for both GPT-5 (*P*=.006) and DeepSeek-V3 (*P*=.01). However, these contrasts reflect the combined addition of MDT and in-context learning and do not isolate the contribution of the MDT layer. In matched comparisons, adding MDT yielded numerically higher AUCs than the corresponding settings without MDT, both without in-context learning (GPT-5: 0.7840 vs 0.7031, *P*=.09; DeepSeek-V3: 0.7458 vs 0.6533, *P*=.22) and with in-context learning (GPT-5: 0.8419 vs 0.8126, *P*=.35; DeepSeek-V3: 0.8003 vs 0.7719, *P*=.55), but none of these differences reached statistical significance. The AUC of GPT-5 with MDT and in-context learning was also not significantly higher than that of the random forest model (P=.18).

**Table 2.** Performance of each large language model for prediction on the internal validation set using all predictor variables.

Model	AUC^a^ (95% CI)	Sensitivity (95% CI)	Specificity (95% CI)	PPV^b^ (95% CI)	NPV^c^ (95% CI)	Accuracy (95% CI)	*F*_1_-score (95% CI)	Brier score (95% CI)
DeepSeek-V3								
No MDT^d^+no context	0.6533 (0.5515-0.7552)	0.7000 (0.2500-0.9630)	0.5650 (0.2249-0.9511)	0.1944 (0.1290-0.4545)	0.9262 (0.8823-0.9832)	0.5826 (0.3216-0.8696)	0.3043 (0.2182-0.4139)	0.1453 (0.1275-0.1623)
MDT+no context	0.7458 (0.6357-0.8560)	0.6667 (0.4346-0.8400)	0.8000 (0.7526-0.9476)	0.3333 (0.2499-0.6002)	0.9412 (0.9000-0.9763)	0.7826 (0.7391-0.9043)	0.4444 (0.3376-0.6217)	0.1064 (0.0834-0.1304)
No MDT+context	0.7719 (0.6796-0.8642)	0.6667 (0.5556-0.9375)	0.8150 (0.5937-0.8756)	0.3509 (0.1935-0.4728)	0.9422 (0.9200-0.9862)	0.7957 (0.6304-0.8479)	0.4598 (0.3103-0.5834)	0.1432 (0.1194-0.1676)
MDT+context	0.8003 (0.6944-0.9062)	0.7000 (0.5312-0.8889)	0.8650 (0.7339-0.9453)	0.4375 (0.2698-0.6667)	0.9505 (0.9176-0.9830)	0.8435 (0.7435-0.9174)	0.5385 (0.3859-0.6977)	0.1091 (0.0909-0.1285)
GPT-5								
No MDT+no context	0.7031 (0.6001-0.8061)	0.7667 (0.3793-0.9355)	0.5950 (0.4950-0.9344)	0.2212 (0.1509-0.4800)	0.9444 (0.8985-0.9835)	0.6174 (0.5348-0.8696)	0.3433 (0.2520-0.4878)	0.1246 (0.1045-0.1443)
MDT+no context	0.7840 (0.6820-0.8860)	0.7000 (0.5416-0.9394)	0.8150 (0.5634-0.9015)	0.3621 (0.2066-0.5385)	0.9477 (0.9167-0.9882)	0.8000 (0.6087-0.8783)	0.4773 (0.3333-0.6316)	0.1151 (0.0982-0.1327)
No MDT+context	0.8126 (0.7216-0.9036)	0.7000 (0.5185-0.8948)	0.8350 (0.6250-0.9436)	0.3889 (0.2208-0.6071)	0.9489 (0.9171-0.9808)	0.8174 (0.6434-0.9043)	0.5000 (0.3422-0.6429)	0.1089 (0.0928-0.1260)
MDT+context	0.8419 (0.7398-0.9440)	0.8000 (0.6296-0.9310)	0.8700 (0.8308-0.9801)	0.4800 (0.3599-0.8333)	0.9667 (0.9375-0.9892)	0.8609 (0.8217-0.9478)	0.6000 (0.4839-, 0.7857)	0.0876 (0.0701-0.1069)

a AUC: area under the receiver operating characteristic curve.

b PPV: positive predictive value.

c NPV: negative predictive value.

d MDT: multidisciplinary team.

Decision curve analysis was performed to examine the net clinical benefit of the LLM-based models, with interpretation focused on the clinically relevant threshold probability range of 0.10 to 0.30 for postoperative early warning and risk stratification. Within this range, the GPT-5+MDT+context configuration showed the most favorable overall net benefit among the GPT-5 settings, particularly around thresholds of 0.20 to 0.30 (Figure S2A in Multimedia Appendix 1). In contrast, the DeepSeek-V3 models showed less stable decision curves, and the MDT+context configuration demonstrated a positive net benefit only within part of the clinically relevant range, without consistently outperforming the no MDT+context setting (Figure S2C in Multimedia Appendix 1). Calibration was also assessed using Brier scores and calibration plots. Among the GPT-5-based configurations, the in-context learning settings showed lower Brier scores than the no-context settings, with MDT+context yielding the lowest Brier score (0.0876) and showing closer agreement between predicted probabilities and observed event rates (Figure S2B in Multimedia Appendix 1). For DeepSeek-V3, the MDT+context configuration also yielded a relatively low Brier score (0.1091), although the calibration curves remained more variable overall (Figure S2D in Multimedia Appendix 1).

**Figure 5.**
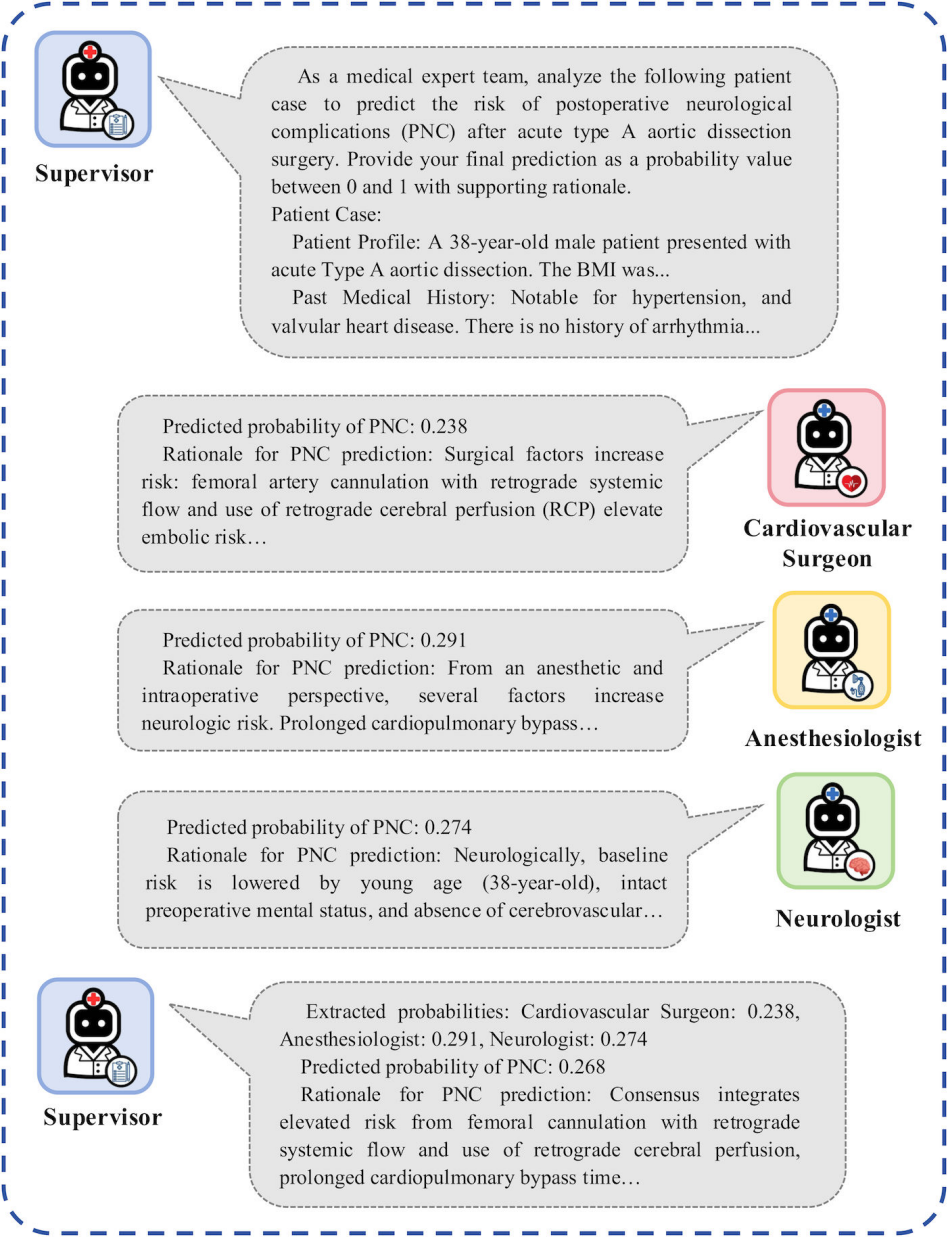
Representative chat-based illustration of the multiagent multidisciplinary team consultation framework. Within the framework, a supervisor agent presents deidentified patient information and coordinates an iterative discussion among 3 role-specialized agents: a cardiovascular surgeon, an anesthesiologist, and a neurologist. Each specialist provides an estimated probability of PNC and a rationale, after which the supervisor summarizes the discussion and outputs the final prediction.

In addition, the dynamic, multiround consultation process among the specialized agents was graphically depicted in a chat-based format in Figure 5. To elucidate the distinct reasoning processes and outputs under each configuration, representative examples for all experimental conditions were provided in the Supplementary Examples in Multimedia Appendix 1. Supplementary Example A illustrates the baseline prediction generated by a single-agent LLM without in-context learning. Supplementary Example B demonstrates the output from the LLM model after in-context learning with population-level statistical summaries. Supplementary Example C provides a transcript of the multiagent discussion conducted without prior in-context learning. Supplementary Example D presents a full 17-round MDT dialog augmented with in-context learning, demonstrating the integration of specialized expertise and background data in evaluating complex risk factors, even in the absence of full consensus. These examples illustrate the collaborative dynamics facilitated by the proposed framework. Nonconsensus events after a maximum of 17 rounds were rare in the internal validation set. Specifically, nonconsensus occurred in 2 of 230 cases (0.87%) for GPT-5 with MDT and in-context learning, whereas no nonconsensus events were observed for other LLM-based prediction models (all 0/230). These findings indicate the high operational efficiency of the simulated MDT framework.

### Word Frequency Analysis of Multiagent Rationales

**Figure 6.**
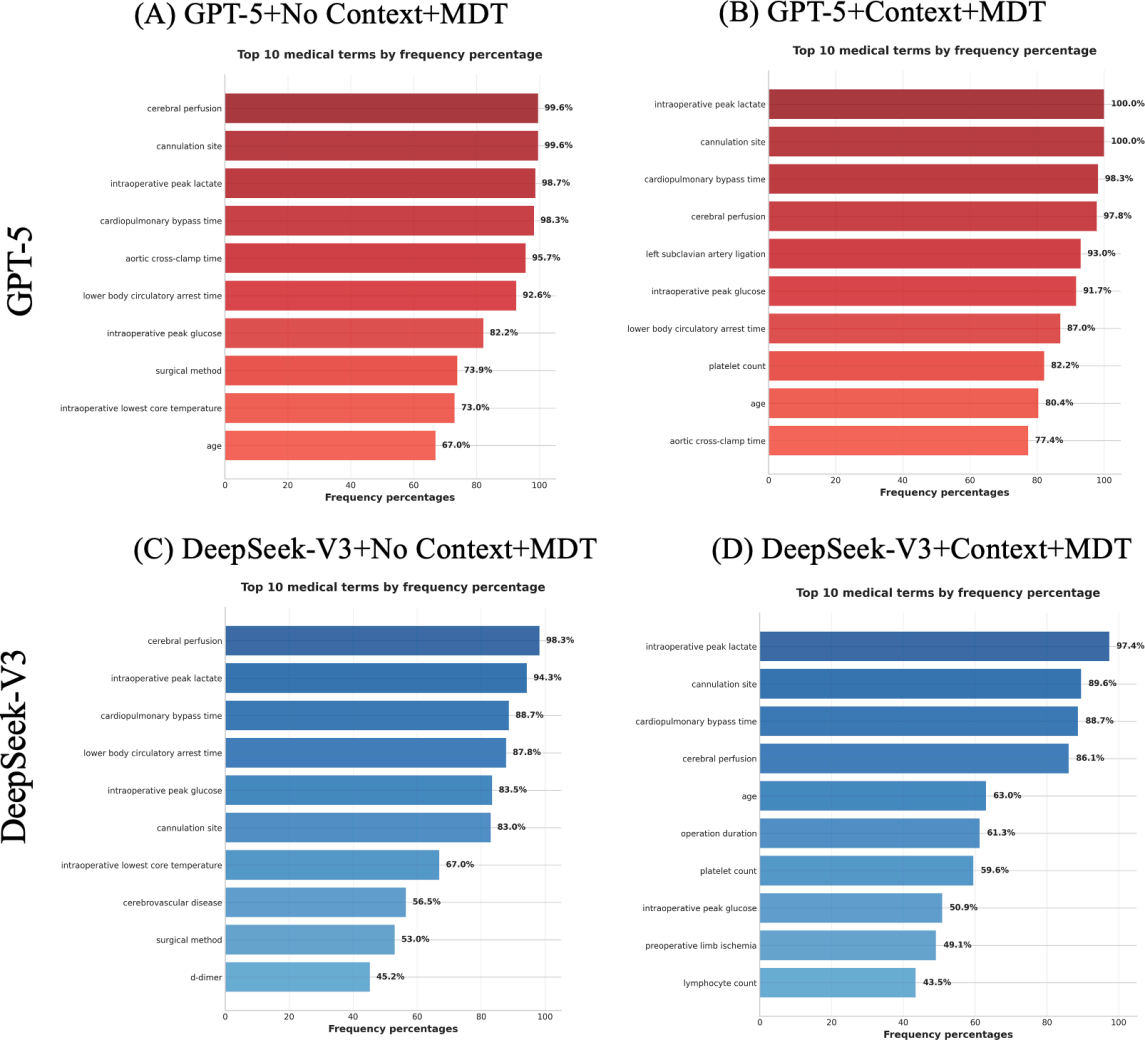
Word frequency analysis of the supervisor agent’s summary rationale. (A) GPT-5+no context+multidisciplinary team (MDT), (B) GPT-5+context+MDT, (C) DeepSeek-V3+no context+MDT, and (D) DeepSeek-V3+context+MDT. Variables are sorted by frequency in descending order.

Word frequency analysis of the summary rationales generated by the supervisor agent in the internal validation set was used as an exploratory analysis of the content emphasized in the model outputs. As shown in Figure 6, the incorporation of in-context learning appeared to shift the emphasis of the generated rationales. In the scenarios without in-context learning (Figures 6A and 6C), the rationales were distributed across a broader range of general patient characteristics. In contrast, when the LLMs were provided with population-level statistical summaries through in-context learning (Figures 6B and 6D), the generated rationales more frequently mentioned variables that were presented as statistically significant in the context, including intraoperative peak lactate level.

### Sensitivity Analysis Using the Selected Variables

To further assess the robustness of the primary findings, we performed a sensitivity analysis in the internal validation set using the same 15 predictors selected by LASSO regression for both ML- and LLM-based models. As summarized in Tables S4 and S5 in Multimedia Appendix 1, restricting the models to the LASSO-selected predictors affected the performance of ML and LLM models differently. Among the ML models, the random forest model showed improved discriminative ability compared with the primary analysis (AUC 0.8308, 95% CI 0.7489‐0.9128), suggesting that the LASSO method could reduce noise and avoid overfitting. In contrast, among the LLM configurations, the use of the MDT framework did not result in a statistically significant improvement in AUC over the corresponding non-MDT settings according to the DeLong test. Although GPT-5 with MDT and in-context learning remained the best-performing LLM configuration with the LASSO-selected variables (AUC 0.8117, 95% CI 0.7144‐0.9089), the incremental benefit of MDT discussion appeared attenuated after variable reduction. This finding suggests that when the patient information is greatly reduced, the additional value of multiagent discussion for LLM-based prediction may be weakened.

### Prospective Validation of LLM Prediction

The prospective validation demonstrated performance trends consistent with the internal validation. For LLMs in Table S6 in Multimedia Appendix 1, GPT-5 with MDT and in-context learning attained the highest AUC. However, for DeepSeek-V3, the MDT with in-context learning configuration yielded slightly lower predictive performance (AUC=0.8009) than the no MDT with in-context learning configuration (AUC=0.8147), suggesting that the multiagent enhancement framework may be model-dependent and influenced by architectural or tuning characteristics. Exploratory pairwise comparisons further showed that adding the MDT framework yielded numerically higher AUCs for GPT-5 both without in-context learning (0.7533 vs 0.7170; *P*=.55) and with in-context learning (0.8280 vs 0.7882; *P*=.21), whereas for DeepSeek-V3, the MDT framework increased AUC in the no-context setting (0.7366 vs 0.6615; *P*=.38) but not in the context setting (0.8009 vs 0.8147; *P*=.83). Nonconsensus events in the prospective cohort were likewise rare, occurring in 1 of 120 cases (0.83%) for DeepSeek-V3 with MDT and in-context learning, while no nonconsensus events were observed in the other MDT configurations (all 0/120). These findings underscore the need for tailored optimization when implementing collaborative LLM frameworks in clinical prediction tasks.

## Discussion

### Principal Findings

In this study, we developed and validated an LLM-based multiagent framework inspired by multidisciplinary collaboration for predicting PNC in patients after ATAAD surgery. In this study setting, GPT-5 combined with both multiagent collaboration and in-context learning achieved the highest discriminative performance, suggesting that the integration of role specialization with population-level statistical context may improve the model’s ability to capture complex risk factors. Notably, we further restricted model inputs to the 15 variables selected by LASSO. The names of these variables are listed in Table S7 in Multimedia Appendix 1. The random forest model numerically outperformed the best-performing GPT-5 configuration (AUC of 0.8308 vs 0.8117), suggesting that traditional ML may derive greater benefit from noise reduction and feature selection when the prediction task is constrained to a smaller set of structured variables. In contrast, among the LLM configurations, the incremental benefit of MDT relative to the corresponding non-MDT setting was attenuated and no longer statistically significant. Although GPT-5 with both multiagent collaboration and in-context learning remained the best-performing LLM configuration in this setting, its advantage over the non-MDT mode was smaller than in the primary analysis. These findings suggest that the added value of multiagent collaboration may depend not only on the model itself but also on the richness of the available input information. When patient information is compressed into a smaller set of key variables, the amount of incremental information available for integration and discussion across roles is correspondingly reduced, thereby weakening the potential benefit of multiround collaboration. At the same time, the relatively low *F*_1_-scores across models should be interpreted in the context of the low incidence of PNC in our cohort, which makes positive predictions intrinsically more difficult and implies that the models may be more suitable for risk stratification and early warning than for establishing a definitive positive diagnosis in isolation. Importantly, this benefit was not consistent across all backbone models. In the prospective validation cohort, GPT-5 maintained the best performance under the multirole setting, whereas the AUC of DeepSeek-V3 decreased from 0.8147 to 0.8009 after the addition of MDT, suggesting that the effect of multiagent collaboration is at least partly model-dependent. Another possible explanation is that the outcome-stratified in-context summaries were derived from the retrospective training cohort and may therefore have been less transportable to the shifted prospective population. This prior population mismatch may have contributed to the performance degradation of DeepSeek-V3 after adding MDT discussion in the in-context learning setting, particularly if the model overweighted retrospective cohort patterns that were less representative of the prospective cohort.

### Comparison With Prior Work

In recent years, LLMs have achieved near-human or even above-human performance on several national medical licensing examinations, suggesting that they encode a substantial amount of biomedical knowledge [38]. However, such benchmarks are typically based on well-structured questions with a single best answer and therefore do not adequately capture the ambiguity, incomplete information, and longitudinal reasoning commonly encountered in real-world clinical practice. Prior studies have shown that when confronted with complex diagnostic cases or real-world prediction tasks, LLMs may still miss key differential diagnoses, and their calibration and awareness of uncertainty remain limited in patient-level clinical decision support [20,39].

To address these limitations, recent work has increasingly explored the use of LLMs within multiagent collaborative frameworks. For example, MedAgents improved zero-shot performance on medical question-answering tasks by organizing specialty-informed agents to debate and aggregate opinions [40], while the clinically inspired multiagent conversational framework proposed by Chen et al [29] also outperformed a single-agent baseline in rare disease diagnosis. At the same time, emerging evidence suggests that the advantage of multiagent collaboration is not stable across all tasks and models. Although multiagent frameworks may achieve better performance in complex, highly structured tasks, many problems can still be handled adequately by simpler single-agent strategies, often at substantially lower computational cost. Recent systematic evaluations of medical agent systems have similarly shown that their overall performance gains over baseline LLMs are modest and not consistently significant across scenarios, indicating that the practical value of multirole collaboration depends on the specific task, the underlying model, and the application context rather than representing a universally superior paradigm [41]. In this study, the relatively small sample size and low event rate led to wide CIs, and most comparisons across LLM configurations did not reach statistical significance. Therefore, the proposed MDT-based framework should be interpreted primarily as a methodological proof-of-concept for structured multiagent risk assessment, and its clinical effectiveness requires further validation in larger prospective cohorts.

Against this background, our findings are broadly consistent with the existing literature. We constructed a multiagent framework composed of a cardiovascular surgeon, anesthesiologist, neurologist, and supervisor, and combined it with in-context learning based on population-level summary statistics. The results indicate that this framework can provide transparent, role-differentiated explanatory reasoning for PNC risk prediction and achieve the best discriminative performance with GPT-5, suggesting that structured collaborative reasoning may offer incremental benefit when paired with a sufficiently capable backbone model. However, the slight performance decline observed in DeepSeek-V3 after adding MDT in the prospective validation also indicates that this added value is model-dependent. One possible explanation is that the multirole framework in this study is built on a single shared backbone model, such that the different roles primarily represent distinct perspectives rather than truly independent information sources or heterogeneous knowledge systems. When the model is already able to make sufficient use of statistical context under the single-agent plus in-context learning setting, additional multiround collaboration may fail to generate meaningful new information and instead introduce coordination costs and informational redundancy, thereby diminishing the potential marginal gain. Taken together, these findings suggest that the primary value of multirole collaboration may lie in improving the structure and interpretability of the reasoning process, whereas gains in predictive performance remain contingent on the specific model and task setting [29,41,42].

In addition, our findings indicate that priming LLMs with population-level summary statistics can effectively ground their reasoning in domain-specific evidence and thereby improve PNC prediction. For resource-constrained clinical settings, this in-context learning strategy offers an accessible route for adapting LLMs to specialized tasks. Unlike fine-tuning, it does not require additional model training or substantial computational resources [43]. Unlike few-shot prompting, it allows more systematic cohort-level information to be introduced within a limited context window [44]. Compared with retrieval-augmented generation, which depends heavily on the quality of the external knowledge base and retrieval performance, this approach is more direct and easier to implement [45]. Thus, in-context learning based on population-level evidence may provide a practical pathway for enhancing the clinical utility of LLMs. However, we must acknowledge that several baseline and operative characteristics differed substantially between the retrospective and prospective cohorts, including arch replacement strategy and pericardial effusion grading. Although these variables were not retained among the 15 predictors selected via LASSO regression, their imbalance may still indicate broader distributional differences between cohorts. These shifts may reflect temporal changes in patient selection, perioperative evaluation, and surgical strategy, but systematic differences between retrospective electronic health record abstraction and prospective departmental data collection cannot be excluded. It should also be emphasized that, although the present multiagent framework draws on the collaborative concept of MDT, it remains, in essence, a role-based reasoning and result-integration process implemented within a single underlying model. Unlike a real clinical MDT, it does not involve physical examination, joint imaging review, longitudinal disease-course assessment, or interactive clinical judgment based on accumulated experience. Accordingly, it is more appropriately regarded as a structured approximation of multidisciplinary decision logic rather than a direct analog of actual MDT consultation. This distinction more accurately reflects the technical nature of the framework and helps clarify both its potential value and its practical boundaries in clinical application.

### Limitations

First, the composite outcome is heterogeneous, including stroke, cerebral hemorrhage, paraplegia, and coma. As these neurological complications may arise from different pathophysiological mechanisms, especially paraplegia versus cerebral events, this outcome definition may have introduced confounding into model development and interpretation. Second, although prospectively validated, the relatively small prospective cohort and limited number of PNC events may have reduced the statistical power of the prospective validation and constrained the stability of performance estimates. In addition, the prospective validation cohort included only patients with complete prespecified predictor variables, which may have introduced selection bias. External validation across different regions, patient populations, and health care systems is therefore needed to further establish its broader applicability. Third, the in-context learning strategy incorporated summary statistics derived from the training cohort, an approach that is not equivalent to standard ML training on individual-level labeled records. The word frequency analysis of the generated rationales was only an exploratory proxy for the content emphasized in the model outputs and should not be interpreted as a direct representation of the models’ internal reasoning or causal decision-making process. Moreover, because the in-context learning prompts explicitly included population-level summary statistics and intergroup *P* values, the increased frequency of certain terms may partly reflect the models’ reiterating variables emphasized in the provided context, rather than independent feature attribution by the LLMs. Fourth, our in-context learning prompt provided population-level summary statistics, including intergroup *P* values, but asked the model to express its final rationale in clinical terms without mentioning statistical significance. Although this design improved the readability and clinician-like style of the outputs, it may have reduced the direct traceability of how statistical information influenced the generated rationale; therefore, these explanations should be interpreted as clinically oriented summaries rather than fully transparent statistical reasoning. Fifth, the consensus criterion in the multiagent discussion was prespecified as a maximum probability range of 0.100 across specialist agents, meaning that the difference between the highest and lowest predicted probabilities could not exceed 10 percentage points. This threshold was chosen as a pragmatic operational criterion to balance near-agreement against unnecessarily prolonged discussion. However, no widely accepted standard currently exists for calibrating consensus thresholds in simulated LLM-MDT deliberation. Therefore, further efforts are needed to explore alternative probability-range cutoffs. Sixth, although MICE was used to handle missing data, the imputed values were averaged to generate a single complete dataset, rather than being analyzed separately with performance estimates pooled via the Rubin rules. This approach may have underestimated the uncertainty associated with missing-data imputation. In addition, imputed values were not explicitly labeled in the patient-specific prompts. Consequently, the LLMs treated them with the same confidence as observed values, which may have affected the models’ reasoning. Seventh, the operating thresholds used to calculate sensitivity, specificity, PPV, NPV, accuracy, and *F*_1_-score were selected by maximizing Youden index within the same internal or prospective validation cohort in which these metrics were evaluated, rather than being prespecified and locked. Consequently, these threshold-dependent point estimates may be optimistically biased and should be interpreted as exploratory, cohort-optimized performance estimates rather than performance at a prespecified clinical operating threshold. This limitation does not affect the threshold-independent AUC comparisons or Brier scores.

### Conclusions

This study developed and validated an MDT-style LLM framework for predicting PNC after ATAAD surgery. The GPT-5 configuration combining multiagent collaboration with population-level in-context learning achieved the highest numerical discrimination among the evaluated LLM settings. However, adding the MDT framework did not produce a statistically significant improvement in AUC over the corresponding single-agent settings, and its incremental predictive value remains unconfirmed. The framework generated structured, role-specific rationales that were broadly consistent with clinically recognized risk factors, supporting its further investigation as a proof-of-concept approach for postoperative risk stratification and early warning. Larger multicenter external validation studies are required before routine clinical implementation.

## Supplementary material

10.2196/91257 Multimedia Appendix 1 Supplementary tables, figures, examples, and data.

10.2196/91257 Checklist 1 TRIPOD+AI checklist.

## Abbreviations

ATAAD: acute type A aortic dissection
AUC: area under the receiver operating characteristic curve
JSON: JavaScript Object Notation
LASSO: least absolute shrinkage and selection operator
LLM: large language model
MDT: multidisciplinary team
MICE: multiple imputation by chained equations
ML: machine learning
NPV: negative predictive value
PNC: postoperative neurological complication
PPV: positive predictive value
SHAP: Shapley Additive Explanations
TRIPOD+AI: Transparent Reporting of a Multivariable Prediction Model for Individual Prognosis or Diagnosis + AI
