# Supplementary material for: A Multidisciplinary Team–Based Large Language Model Framework for Predicting Postoperative Neurological Complications in Acute Type A Aortic Dissection: Model Development and Validation Study
Source: J Med Internet Res. 2026 Aug 18;28:e91257. doi: 10.2196/91257 (PMC13484818; doi:10.2196/91257)
Supplement: Supplementary file 2 [file jmir-v28-e91257-s001.docx]

**Supplementary Materials**

**Contents**

**Supplementary Table 1.** Characteristics for patients with and without PNC following ATAAD surgery in the retrospective cohort.

**Supplementary Table 2.** The comparison of characteristics between the retrospective cohort and prospective validation cohort.

**Supplementary Table 3.** Summary of within-patient variability across repeated GPT-5 inference runs under four application settings.

**Supplementary Table 4.** Performance of each ML model for prediction using 15 predictors selected via LASSO regression.

**Supplementary Table 5.** Performance of each LLM model for prediction using 15 predictors selected via LASSO regression.

**Supplementary Table 6.** Performance of each LLM model for prediction in the prospective validation cohort using all predictor variables.

**Supplementary Table 7.** Mapping of the 15 predictors between JSON variable names and full clinical names selected via LASSO regression.

**Supplementary Figure 1.** Repeated predicted probabilities and distribution of within-patient variability metrics across 5 independent GPT-5 runs under four application settings.

**Supplementary Figure 2.** Decision curve analysis and calibration plots of the LLM models: (A) Decision curve analysis of the four GPT-5-based prediction modes; (B) Calibration plots of the four GPT-5-based prediction modes; (C) Decision curve analysis of the four DeepSeek-V3-based prediction modes; (D) Calibration plots of the four DeepSeek-V3-based prediction modes.

**Supplementary Example A:** Output of a single-agent LLM prediction without in-context learning.

**Supplementary Example B:** Output of a single-agent LLM prediction with in-context learning.

**Supplementary Example C:** Transcript of a multi-agent discussion (MDT without in-context learning).

**Supplementary Example D:** Transcript of a multi-agent discussion reaching the maximum 17 rounds (MDT with in-context learning).

**Supplementary Data 1**. Complete JSON structure of all candidate variables used for in-context learning.

**Supplementary Data 2**. Complete JSON structure of the 15 LASSO-selected predictors used for in-context learning.

**Supplementary Table 1.** Characteristics for patients with and without PNC following ATAAD surgery in the retrospective cohort.

| **Variable** | **Missing** | **No PNC group**  **(N = 664)** | **PNC group**  **(N = 99)** | **P value** |
| --- | --- | --- | --- | --- |
| **Demographic** |  |  |  |  |
| age (years) | 0 (0%) | 51 (43, 59) | 57 (49.5, 65) | <.001 |
| bmi (kg/m²) | 0 (0%) | 25.23 (22.66, 27.89) | 24.64 (22.55, 27.37) | .588 |
| sex (male), n(%) | 0 (0%) | 509 (76.7%) | 73 (73.7%) | .524 |
| smoking history, n(%) | 0 (0%) | 261 (39.3%) | 37 (37.4%) | .713 |
| alcoholic history, n(%) | 0 (0%) | 217 (32.7%) | 23 (23.2%) | .059 |
| **Past medical history** |  |  |  |  |
| hypertension, n(%) | 0 (0%) | 472 (71.1%) | 69 (69.7%) | .777 |
| arrhythmia, n(%) | 0 (0%) | 17 (2.6%) | 3 (3%) | .736 |
| diabetes, n(%) | 0 (0%) | 18 (2.7%) | 6 (6.1%) | .112 |
| marfan syndrome, n(%) | 0 (0%) | 21 (3.2%) | 0 (0%) | .095 |
| renal insufficiency, n(%) | 0 (0%) | 36 (5.4%) | 11 (11.1%) | .028 |
| coronary heart disease, n(%) | 0 (0%) | 12 (1.8%) | 3 (3%) | .429 |
| stroke, n(%) | 0 (0%) | 17 (2.6%) | 8 (8.1%) | .009 |
| carotid atherosclerosis, n(%) | 0 (0%) | 3 (0.5%) | 0 (0%) | 1 |
| thrombosis, n(%) | 0 (0%) | 3 (0.5%) | 0 (0%) | 1 |
| valvular heart disease, n(%) | 0 (0%) | 73 (11%) | 10 (10.1%) | .79 |
| COPD, n(%) | 0 (0%) | 31 (4.7%) | 3 (3%) | .607 |
| thyroid disease, n(%) | 0 (0%) | 9 (1.4%) | 1 (1%) | 1 |
| previous surgical history, n(%) | 0 (0%) | 103 (15.5%) | 12 (12.1%) | .379 |
| **Preoperative laboratory findings** |  |  |  |  |
| Complete Blood Count |  |  |  |  |
| hemoglobin (g/L) | 2 (0.3%) | 128 (113, 140) | 124 (106, 137.5) | .171 |
| WBC (10^9^/L) | 2 (0.3%) | 10.92 (8.71, 13.58) | 11.85 (9.31, 14.2) | .031 |
| platelets (10^9^/L) | 2 (0.3%) | 149 (108, 189.25) | 136 (98, 169.5) | .018 |
| neutrophil percentage (%) | 2 (0.3%) | 82.4 (76.97, 87) | 84.7 (79.45, 89.25) | .002 |
| absolute neutrophil count (10^9^/L) | 2 (0.3%) | 8.91 (6.85, 11.6) | 10.19 (7.7, 11.88) | .012 |
| lymphocyte (10^9^/L) | 2 (0.3%) | 0.95 (0.68, 1.31) | 0.86 (0.58, 1.13) | .005 |
| monocyte (10^9^/L) | 2 (0.3%) | 0.84 (0.6, 1.13) | 0.87 (0.54, 1.15) | .933 |
| Coagulation |  |  |  |  |
| pt (s) | 4 (0.5%) | 11.7 (11.1, 12.4) | 11.8 (11.15, 12.6) | .485 |
| inr | 4 (0.5%) | 1.04 (0.99, 1.11) | 1.06 (1, 1.12) | .229 |
| fibrinogen (g/L) | 6 (0.8%) | 2.89 (2.07, 4.42) | 2.36 (1.77, 3.5) | .005 |
| d-dimer (mg/L FEU) | 58 (7.6%) | 6.2 (2.57, 13.35) | 9.52 (4.94, 26.74) | <.001 |
| Thyroid Function |  |  |  |  |
| tsh (mIU/L) | 83 (10.9%) | 1.22 (0.64, 2.11) | 1.36 (0.76, 2.45) | .140 |
| t3 (nmol/L) | 85 (11.1%) | 1.3 (1.06, 1.54) | 1.33 (1.04, 1.54) | .751 |
| ft3 (pmol/L) | 83 (10.9%) | 3.72 (3.13, 4.26) | 3.6 (2.98, 4.25) | .297 |
| t4 (nmol/L) | 87 (11.4%) | 83.7 (72.07, 95.43) | 84.2 (73.4, 98.57) | .304 |
| ft4 (pmol/L) | 86 (11.3%) | 15.1 (13.5, 16.61) | 15.4 (13.73, 17.38) | .234 |
| Chemistry&Cardiac Markers |  |  |  |  |
| bilirubin (μmol/L) | 3 (0.4%) | 16.15 (11.3, 22.75) | 15.6 (11.05, 21.75) | .827 |
| hdl (mmol/L) | 3 (0.4%) | 1.11 (0.9, 1.34) | 1.12 (0.94, 1.45) | .219 |
| ldl (mmol/L) | 3 (0.4%) | 2.24 (1.68, 2.81) | 2.36 (1.88, 2.75) | .605 |
| triglycerides (mmol/L) | 3 (0.4%) | 1.17 (0.85, 1.64) | 1.15 (0.9, 1.67) | .676 |
| total cholesterol (mmol/L) | 3 (0.4%) | 3.84 (3.21, 4.47) | 3.98 (3.28, 4.4) | .573 |
| uric acid (μmol/L) | 3 (0.4%) | 358 (278, 442.5) | 391 (301.5, 481.5) | .068 |
| creatinine (μmol/L) | 3 (0.4%) | 90 (71, 116) | 100 (78, 135) | .030 |
| preoperative fasting blood glucose (mmol/L) | 3 (0.4%) | 6.84 (5.82, 7.99) | 7.46 (6.67, 9.33) | <.001 |
| albumin (g/L) | 3 (0.4%) | 39.2 (36.4, 41.8) | 39 (36.2, 42.2) | .834 |
| alt (IU/L) | 3 (0.4%) | 21 (14, 38) | 18 (14, 32.5) | .266 |
| ast (IU/L) | 3 (0.4%) | 22 (16, 36) | 25 (16, 46) | .146 |
| preoperative myoglobin (ng/mL) | 9 (1.18%) | 39.48 (21.76, 92.01) | 78.63 (37.72, 230.5) | <.001 |
| bnp (ng/L) | 5 (0.7%) | 415 (167, 956) | 483 (256, 1134.5) | .043 |
| crp (mg/L) | 74 (9.7%) | 57.8 (14.5, 116) | 27.2 (8.59, 104) | .032 |
| **Preoperative imaging&Status** |  |  |  |  |
| Blood Gas |  |  |  |  |
| preoperative PaO2 (mmHg) | 81 (10.6%) | 94.45 (76, 119.85) | 98.2 (75.4, 124.75) | .704 |
| preoperative PaCO2 (mmHg) | 82 (10.7%) | 36.2 (32.1, 40.3) | 36.8 (31.75, 42) | .311 |
| Anatomy&Status |  |  |  |  |
| preoperative hypotension, n(%) | 0 (0%) | 91 (13.7%) | 20 (20.2%) | .087 |
| preoperative alteration of consciousness state, n(%) | 0 (0%) | 70 (10.5%) | 16 (16.2%) | .099 |
| preoperative limb ischemia, n(%) | 0 (0%) | 107 (16.1%) | 32 (32.3%) | <.001 |
| entry tear, n(%) | 0 (0%) |  |  |  |
| ascending aorta |  | 464 (69.9%) | 79 (79.8%) | .278 |
| arcus aortae |  | 156 (23.5%) | 16 (16.2%) |  |
| descending aorta |  | 29 (4.4%) | 3 (3%) |  |
| unknown |  | 15 (2.3%) | 1 (1%) |  |
| retrograde dissection, n(%) | 0 (0%) | 17 (2.6%) | 5 (5.1%) | .189 |
| sma, n(%) | 0 (0%) |  |  |  |
| sma originating from the true cavity |  | 482 (72.6%) | 72 (72.7%) | .915 |
| partial involvement of the sma |  | 155 (23.3%) | 24 (24.2%) |  |
| severe involvement of the sma |  | 27 (4.1%) | 3 (3%) |  |
| bovine arch, n(%) | 0 (0%) | 6 (0.9%) | 4 (4%) | .030 |
| pericardial effusion volume, n(%) | 0 (0%) |  |  |  |
| normal |  | 62 (9.3%) | 7 (7.1%) | .837 |
| few |  | 462 (69.6%) | 73 (73.7%) |  |
| medium |  | 104 (15.7%) | 14 (14.1%) |  |
| large |  | 36 (5.4%) | 5 (5.1%) |  |
| **Operative details** |  |  |  |  |
| Procedure |  |  |  |  |
| arch replacement, n(%) | 0 (0%) |  |  |  |
| no |  | 47 (7.1%) | 7 (7.1%) | .232 |
| hemi arch replacement |  | 126 (19%) | 26 (26.3%) |  |
| total arch replacement |  | 491 (73.9%) | 66 (66.7%) |  |
| aortic root procedure, n(%) | 0 (0%) |  |  |  |
| aortic root preserved |  | 81 (12.2%) | 8 (8.1%) | .441 |
| aortic root reconstruction |  | 331 (49.8%) | 54 (54.5%) |  |
| aortic root replacement |  | 252 (38%) | 37 (37.4%) |  |
| coronary artery bypass grafting, n(%) | 0 (0%) | 57 (8.6%) | 11 (11.1%) | .410 |
| ligation of sca without subsequent reconstruction, n(%) | 0 (0%) | 29 (4.4%) | 10 (10.1%) | .016 |
| Parameters |  |  |  |  |
| operation duration (h) | 0 (0%) | 6.9 (6.1, 8) | 7.5 (6.5, 8.3) | .001 |
| lowest temperature (℃) | 0 (0%) | 24 (23.3, 24.7) | 24 (23.3, 24.9) | .704 |
| cpb time (min) | 0 (0%) | 191.5 (164, 235) | 209 (177.5, 257.5) | .007 |
| acc time (min) | 0 (0%) | 141 (113.75, 175) | 152 (113.5, 192.5) | .154 |
| lower body circulatory arrest time (min) | 47 (6.2%) | 25 (19, 31) | 25 (19.5, 32.5) | .649 |
| Perfusion&Transfusion |  |  |  |  |
| cannulation site, n(%) | 0 (0%) |  |  |  |
| axillary artery |  | 116 (17.5%) | 12 (12.1%) | .105 |
| arteria cruralis |  | 508 (76.5%) | 77 (77.8%) |  |
| axillary artery+arteria cruralis |  | 22 (3.3%) | 3 (3%) |  |
| other |  | 18 (2.7%) | 7 (7.1%) |  |
| cerebral perfusion, n(%) | 0 (0%) |  |  |  |
| no |  | 78 (11.7%) | 15 (15.2%) | .136 |
| sequential perfusion,unilateral |  | 430 (64.8%) | 56 (56.6%) |  |
| sequential perfusion,bilateral |  | 76 (11.4%) | 9 (9.1%) |  |
| retrograde perfusion |  | 80 (12%) | 19 (19.2%) |  |
| intraoperative plasma transfusion (mL) | 0 (0%) | 0 (0, 0) | 0 (0, 400) | .012 |
| intraoperative RBC transfusion (units) | 0 (0%) | 0 (0, 2) | 0 (0, 2.25) | .012 |
| intraoperative platelets transfusion (units) | 0 (0%) | 2.0 (1.0, 2.0) | 2.0 (1.0, 2.0) | .006 |
| fluid volume (mL) | 0 (0%) | 1600 (1200, 2062.5) | 1800 (1500, 2300) | .026 |
| max glucose intraoperation (mmol/L) | 0 (0%) | 10.2 (8.57, 12.22) | 11.2 (9.15, 13.5) | .038 |
| max lactate intraoperation (mmol/L) | 4 (0.5%) | 5.2 (3.8, 7.9) | 7.8 (4.65, 12.8) | <.001 |

**Supplementary Table 2.** The comparison of characteristics between the retrospective cohort and prospective validation cohort.

| **Variable** | **Retrospective cohort**  **(n=763)** | **Prospective validation cohort**  **(n=120)** | **P value** |
| --- | --- | --- | --- |
| **Demographic** |  |  |  |
| age (years) | 51.00 (44.00, 59.00) | 51.00 (44.25, 58.75) | .688 |
| bmi (kg/m²) | 25.18 (22.65, 27.77) | 25.24 (22.85, 27.76) | .957 |
| sex (male), n(%) | 582 (76.3) | 84 (70) | .170 |
| smoking history, n(%) | 298 (39.1) | 16 (13.3) | <.001 |
| alcoholic history, n(%) | 240 (31.5) | 25 (20.8) | .024 |
| **Past medical history** |  |  |  |
| hypertension, n(%) | 541 (70.9) | 91 (75.8) | .315 |
| arrhythmia, n(%) | 20 (2.6) | 3 (2.5) | 1 |
| diabetes, n(%) | 24 (3.1) | 2 (1.7) | .548 |
| marfan syndrome, n(%) | 21 (2.8) | 6 (5.0) | .296 |
| renal insufficiency, n(%) | 47 (6.2) | 2 (1.7) | .074 |
| coronary heart disease, n(%) | 15 (2.0) | 4 (3.3) | .534 |
| stroke, n(%) | 25 (3.3) | 5 (4.2) | .819 |
| carotid atherosclerosis, n(%) | 3 (0.4) | 3 (2.5) | .044 |
| thrombosis, n(%) | 3 (0.4) | 2 (1.7) | .283 |
| valvular heart disease, n(%) | 83 (10.9) | 22 (18.3) | .028 |
| COPD, n(%) | 34 (4.5) | 3 (2.5) | .454 |
| thyroid disease, n(%) | 10 (1.3) | 2 (1.7) | 1 |
| previous surgical history, n(%) | 115 (15.1) | 16 (13.3) | .719 |
| **Preoperative laboratory findings** |  |  |  |
| Complete Blood Count |  |  |  |
| hemoglobin (g/L) | 128.00 (112.00, 140.00) | 120.50 (109.75, 138.25) | .121 |
| WBC (10^9^/L) | 11.03 (8.84, 13.73) | 9.16 (6.84, 11.62) | <.001 |
| platelets (10^9^/L) | 147.00 (106.50, 186.50) | 157.00 (108.00, 215.00) | .115 |
| neutrophil percentage (%) | 82.60 (77.20, 87.25) | 77.25 (65.50, 82.10) | <.001 |
| absolute neutrophil count (10^9^/L) | 9.07 (6.99, 11.62) | 6.73 (4.45, 9.74) | <.001 |
| lymphocyte (10^9^/L) | 0.94 (0.66, 1.30) | 1.12 (0.76, 1.67) | <.001 |
| monocyte (10^9^/L) | 0.84 (0.59, 1.13) | 0.72 (0.49, 1.05) | .014 |
| Coagulation |  |  |  |
| pt (s) | 11.70 (11.10, 12.40) | 12.40 (11.40, 14.03) | <.001 |
| inr | 1.05 (0.99, 1.11) | 1.07 (1.00, 1.22) | .008 |
| fibrinogen (g/L) | 2.83 (2.03, 4.30) | 3.30 (2.47, 4.56) | .034 |
| d-dimer (mg/L FEU) | 6.71 (2.74, 14.23) | 4.03 (1.68, 8.44) | <.001 |
| Thyroid Function |  |  |  |
| tsh (mIU/L) | 1.23 (0.66, 2.14) | 1.63 (0.84, 2.80) | .002 |
| t3 (nmol/L) | 1.3 (1.06, 1.54) | 1.48 (1.23, 1.71) | <.001 |
| ft3 (pmol/L) | 3.71 (3.10, 4.26) | 3.82 (3.33, 4.50) | .018 |
| t4 (nmol/L) | 83.80 (72.10, 95.70) | 93.25 (81.53, 105.00) | <.001 |
| ft4 (pmol/L) | 15.20 (13.50, 16.70) | 15.55 (14.05, 16.88) | .154 |
| Chemistry&Cardiac Markers |  |  |  |
| bilirubin (μmol/L) | 16.10 (11.30, 22.55) | 13.55 (9.38, 19.83) | .001 |
| hdl (mmol/L) | 1.11 (0.91, 1.34) | 1.02 (0.80, 1.26) | .014 |
| ldl (mmol/L) | 2.27 (1.69, 2.80) | 2.09 (1.51, 2.83) | .273 |
| triglycerides (mmol/L) | 1.17 (0.85, 1.65) | 1.29 (0.94, 1.81) | .111 |
| total cholesterol (mmol/L) | 3.85 (3.22, 4.47) | 3.83 (3.04, 4.70) | .947 |
| uric acid (μmol/L) | 362.00 (281.50, 448.00) | 371.00 (272.25, 438.00) | .868 |
| creatinine (μmol/L) | 91.00 (72.00, 117.00) | 84.50 (67.00, 111.25) | .056 |
| preoperative fasting blood glucose (mmol/L) | 6.95 (5.93, 8.11) | 6.46 (4.94, 7.73) | <.001 |
| albumin (g/L) | 39.20 (36.40, 41.80) | 39.10 (36.10, 41.68) | .431 |
| alt (IU/L) | 20.00 (14.00, 37.50) | 19.00 (13.00, 36.75) | .650 |
| ast (IU/L) | 22.00 (16.00, 37.50) | 20.00 (16.00, 29.00) | .094 |
| preoperative myoglobin (ng/mL) | 41.68 (22.91, 103.90) | 42.18 (25.50, 96.53) | .745 |
| bnp (ng/L) | 421.00 (176.00, 976.50) | 418.50 (176.00, 1340.50) | .391 |
| crp (mg/L) | 55.40 (13.45, 115.00) | 25.65 (7.12, 79.83) | <.001 |
| **Preoperative imaging&Status** |  |  |  |
| Blood Gas |  |  |  |
| preoperative PaO2 (mmHg) | 95.10 (76.00, 121.80) | 85.85 (73.50, 105.05) | .007 |
| preoperative PaCO2 (mmHg) | 36.20 (32.00, 40.40) | 39.85 (36.10, 43.87) | <.001 |
| Anatomy&Status |  |  |  |
| preoperative hypotension, n(%) | 111 (14.5) | 13 (10.8) | .343 |
| preoperative alteration of consciousness state, n(%) | 86 (11.3) | 24 (20.0) | .011 |
| preoperative limb ischemia, n(%) | 139 (18.2) | 26 (21.7) | .438 |
| entry tear, n(%) |  |  |  |
| ascending aorta | 543 (71.2) | 97 (80.8) | .044 |
| arcus aortae | 172 (22.5) | 20 (16.7) |  |
| descending aorta | 32 (4.2) | 0 (0.0) |  |
| unknown | 16 (2.1) | 3 (2.5) |  |
| retrograde dissection, n(%) | 22 (2.9) | 11 (9.2) | .002 |
| sma, n(%) |  |  |  |
| sma originating from the true cavity | 554 (72.6) | 53 (44.2) | <.001 |
| partial involvement of the sma | 179 (23.5) | 34 (28.3) |  |
| severe involvement of the sma | 30 (3.9) | 33 (27.5) |  |
| bovine arch, n(%) | 10 (1.3) | 0 (0.0) | .425 |
| pericardial effusion volume, n(%) |  |  |  |
| normal | 69 (9.0) | 79 (65.8) | <.001 |
| few | 535 (70.1) | 14 (11.7) |  |
| medium | 118 (15.5) | 13 (10.8) |  |
| large | 41 (5.4) | 14 (11.7) |  |
| **Operative details** |  |  |  |
| Procedure |  |  |  |
| arch replacement, n(%) |  |  |  |
| no | 54 (7.1) | 40 (33.3) | <.001 |
| hemi arch replacement | 152 (19.9) | 9 (7.5) |  |
| total arch replacement | 557 (73.0) | 71 (59.2) |  |
| aortic root procedure, n(%) |  |  |  |
| aortic root preserved | 89 (11.7) | 36 (30.0) | <.001 |
| aortic root reconstruction | 385 (50.5) | 36 (30.0) |  |
| aortic root replacement | 289 (37.9) | 48 (40.0) |  |
| coronary artery bypass grafting, n(%) | 68 (8.9) | 9 (7.5) | .737 |
| ligation of sca without subsequent reconstruction, n(%) | 39 (5.1) | 0 (0.0) | .022 |
| Parameters |  |  |  |
| operation duration (h) | 7 (6.12, 8.00) | 6.04 (3.83, 7.18) | <.001 |
| lowest temperature (℃) | 24.00 (23.30, 24.70) | 23.00 (23.00, 24.00) | <.001 |
| cpb time (min) | 193.00 (165.00, 235.50) | 187.00 (160.75, 225.00) | .237 |
| acc time (min) | 142.00 (113.50, 177.50) | 140.50 (111.75, 178.00) | .903 |
| lower body circulatory arrest time (min) | 25.00 (19.00, 31.00) | 25.00 (22.00, 29.00) | .270 |
| Perfusion&Transfusion |  |  |  |
| cannulation site, n(%) |  |  |  |
| axillary artery | 128 (16.8) | 9 (7.5) | <.001 |
| arteria cruralis | 585 (76.7) | 73 (60.8) |  |
| axillary artery+arteria cruralis | 25 (3.3) | 12 (10.0) |  |
| other | 25 (3.3) | 26 (21.7) |  |
| cerebral perfusion, n(%) |  |  |  |
| no | 93 (12.2) | 49 (40.8) | <.001 |
| sequential perfusion,unilateral | 486 (63.7) | 56 (46.7) |  |
| sequential perfusion,bilateral | 85 (11.1) | 14 (11.7) |  |
| retrograde perfusion | 99 (13.0) | 1 (0.8) |  |
| intraoperative plasma transfusion (mL) | 0.00 (0.00, 200.00) | 400.00 (0.00, 600.00) | <.001 |
| intraoperative RBC transfusion (units) | 0.00 (0.00, 2.00) | 0.00 (0.00, 2.00) | .891 |
| intraoperative platelets transfusion (units) | 2.00 (1.00, 2.00) | 1.00 (1.00, 2.00) | .002 |
| fluid volume (mL) | 1600.00 (1200.00, 2100.00) | 1125.00 (1000.00, 1412.50) | <.001 |
| max glucose intraoperation (mmol/L) | 10.30 (8.60, 12.40) | 8.90 (7.68, 10.43) | <.001 |
| max lactate intraoperation (mmol/L) | 5.50 (3.90, 8.50) | 5.20 (3.40, 8.50) | .298 |

**Supplementary Table 3.** Summary of within-patient variability across repeated GPT-5 inference runs under four application settings.

| **Model** | **mean_range** | **median_range** | **max_range** | **mean_sd** | **median_sd** | **mean_iqr** | **median_iqr** |
| --- | --- | --- | --- | --- | --- | --- | --- |
| No MDT + No Context | 0.037 | 0.040 | 0.080 | 0.016 | 0.015 | 0.020 | 0.020 |
| MDT + No Context | 0.054 | 0.055 | 0.100 | 0.023 | 0.023 | 0.028 | 0.025 |
| No MDT + Context | 0.042 | 0.040 | 0.090 | 0.018 | 0.015 | 0.022 | 0.020 |
| MDT + Context | 0.036 | 0.029 | 0.110 | 0.015 | 0.013 | 0.018 | 0.012 |

**Supplementary Table 4.** Performance of each ML model for prediction using 15 predictors selected via LASSO regression.

| **Model** | **AUC** | **Sensitivity** | **Specificity** | **PPV** | **NPV** | **Accuracy** | **F1 score** | **Brier score** |
| --- | --- | --- | --- | --- | --- | --- | --- | --- |
| Random Forest | 0.8308  (0.7489, 0.9128) | 0.9000  (0.6388, 1.0000) | 0.6750  (0.6269, 0.9246) | 0.2935  (0.2282, 0.6102) | 0.9783  (0.9390, 1.0000) | 0.7043  (0.6652, 0.9043) | 0.4426  (0.3651, 0.6668) | 0.0955  (0.0685, 0.1229) |
| XGBoost | 0.7765  (0.6745, 0.8785) | 0.7333  (0.5555, 0.8919) | 0.8050  (0.7098, 0.9001) | 0.3607  (0.2500, 0.5185) | 0.9527  (0.9186, 0.9832) | 0.7957  (0.7215, 0.8783) | 0.4835  (0.3571, 0.6237) | 0.0961  (0.0683, 0.1249) |
| Naive Bayes | 0.7485  (0.6385, 0.8585) | 0.6667  (0.4800, 0.8621) | 0.8250  (0.6073, 0.9246) | 0.3636  (0.2272, 0.5527) | 0.9429  (0.9086, 0.9771) | 0.8043  (0.6304, 0.8870) | 0.4706  (0.3359, 0.6207) | 0.1253  (0.0870, 0.1673) |
| Logistic Regression | 0.7152  (0.6059, 0.8245) | 0.6000  (0.3667, 0.8667) | 0.7850  (0.5968, 0.9400) | 0.2951  (0.1772, 0.5485) | 0.9290  (0.8922, 0.9742) | 0.7609  (0.6174, 0.8826) | 0.3956  (0.2791, 0.5517) | 0.0995  (0.0698, 0.1293) |

**Supplementary Table 5.** Performance of each LLM model for prediction using 15 predictors selected via LASSO regression

| **Model** | **AUC** | **Sensitivity** | **Specificity** | **PPV** | **NPV** | **Accuracy** | **F1 score** | **Brier score** |
| --- | --- | --- | --- | --- | --- | --- | --- | --- |
| **DeepSeek-V3** |  |  |  |  |  |  |  |  |
| No MDT + No Context | 0.6820  (0.5788, 0.7852) | 0.7667  (0.2999, 0.9394) | 0.5450  (0.4109, 0.9657) | 0.2018  (0.1389, 0.6155) | 0.9397  (0.8844, 0.9826) | 0.5739  (0.4652, 0.8957) | 0.3194  (0.2346, 0.5000) | 0.1520  (0.1337, 0.1710) |
| MDT + No Context | 0.6727  (0.5709, 0.7744) | 0.5667  (0.3704, 1.0000) | 0.7250  (0.2390, 0.9406) | 0.2361  (0.1329, 0.5000) | 0.9177  (0.8913, 1.0000) | 0.7043  (0.3304, 0.8696) | 0.3333  (0.2322, 0.4865) | 0.1442  (0.1266, 0.1613) |
| No MDT + Context | 0.7724  (0.6744, 0.8704) | 0.7667  (0.5600, 0.9167) | 0.7400  (0.6834, 0.8912) | 0.3067  (0.2131, 0.4914) | 0.9548  (0.9182, 0.9869) | 0.7435  (0.6913, 0.8609) | 0.4381  (0.3243, 0.5834) | 0.1388  (0.1176, 0.1628) |
| MDT + Context | 0.7910  (0.6897, 0.8923) | 0.6667  (0.5666, 0.9189) | 0.8600  (0.6844, 0.9113) | 0.4167  (0.2353, 0.5715) | 0.9451  (0.9206, 0.9869) | 0.8348  (0.7000, 0.8871) | 0.5128  (0.3594, 0.6532) | 0.1091  (0.0909, 0.1284) |
| **GPT-5** |  |  |  |  |  |  |  |  |
| No MDT + No Context | 0.7251  (0.6195, 0.8306) | 0.6333  (0.4242, 0.8501) | 0.7850  (0.6561, 0.9463) | 0.3065  (0.1964, 0.5626) | 0.9345  (0.9017, 0.9749) | 0.7652  (0.6651, 0.8826) | 0.4130  (0.2941, 0.5477) | 0.1349  (0.1185, 0.1509) |
| MDT + No Context | 0.7435  (0.6464, 0.8406) | 0.6667  (0.4800, 0.8848) | 0.8000  (0.6244, 0.8855) | 0.3333  (0.2075, 0.4694) | 0.9412  (0.9064, 0.9778) | 0.7826  (0.6435, 0.8522) | 0.4444  (0.3146, 0.5743) | 0.1278  (0.1105, 0.1442) |
| No MDT + Context | 0.8041  (0.7135, 0.8946) | 0.7333  (0.4848, 0.8966) | 0.7950  (0.7512, 0.9534) | 0.3492  (0.2499, 0.6667) | 0.9521  (0.9146, 0.9826) | 0.7870  (0.7478, 0.9174) | 0.4731  (0.3636, 0.6774) | 0.1023  (0.0820, 0.1229) |
| MDT + Context | 0.8117  (0.7144, 0.9089) | 0.6667  (0.5000, 0.8621) | 0.9250  (0.8010, 0.9659) | 0.5714  (0.3226, 0.7586) | 0.9487  (0.9188, 0.9800) | 0.8913  (0.7912, 0.9348) | 0.6154  (0.4383, 0.7458) | 0.1014  (0.0818, 0.1204) |

**Supplementary Table 6.** Performance of each LLM model for prediction in the prospective validation cohort using all predictor variables.

| **Model** | **AUC** | **Sensitivity** | **Specificity** | **PPV** | **NPV** | **Accuracy** | **F1 score** | **Brier score** |
| --- | --- | --- | --- | --- | --- | --- | --- | --- |
| **DeepSeek-V3** |  |  |  |  |  |  |  |  |
| No MDT + No Context | 0.6615  (0.5403, 0.7828) | 0.6316  (0.4761, 1.0000) | 0.6436  (0.2061, 0.8558) | 0.2500  (0.1429, 0.4400) | 0.9028  (0.8706, 1.0000) | 0.6417  (0.3250, 0.8083) | 0.3582  (0.2478, 0.5201) | 0.1625  (0.1343, 0.1924) |
| MDT + No Context | 0.7366  (0.6064, 0.8668) | 0.6316  (0.3913, 1.0000) | 0.7822  (0.2811, 0.9596) | 0.3529  (0.1852, 0.7273) | 0.9186  (0.8651, 1.0000) | 0.7583  (0.4000, 0.9000) | 0.4528  (0.3077, 0.6667) | 0.1423  (0.1190, 0.1675) |
| No MDT + Context | 0.8147  (0.7269, 0.9026) | 0.7895  (0.6667, 1.0000) | 0.7921  (0.5252, 0.8641) | 0.4167  (0.2319, 0.5814) | 0.9524  (0.9186, 1.0000) | 0.7917  (0.5917, 0.8583) | 0.5455  (0.3678, 0.6875) | 0.1343  (0.1029, 0.1660) |
| MDT + Context | 0.8009  (0.6880, 0.9139) | 0.6316  (0.4736, 1.0000) | 0.8812  (0.4951, 0.9706) | 0.5000  (0.2142, 0.7895) | 0.9271  (0.8800, 1.0000) | 0.8417  (0.5663, 0.9250) | 0.5581  (0.3478, 0.7556) | 0.1182  (0.0940, 0.1443) |
| **GPT-5** |  |  |  |  |  |  |  |  |
| No MDT + No Context | 0.7170  (0.6129, 0.8212) | 0.8947  (0.6499, 1.0000) | 0.5545  (0.3660, 0.8000) | 0.2742  (0.1731, 0.4375) | 0.9655  (0.9091, 1.0000) | 0.6083  (0.4665, 0.7917) | 0.4198  (0.2884, 0.5745) | 0.1365  (0.1079, 0.1664) |
| MDT + No Context | 0.7533  (0.6405, 0.8660) | 0.9474  (0.5833, 1.0000) | 0.5347  (0.4622, 0.9001) | 0.2769  (0.1852, 0.5500) | 0.9818  (0.9111, 1.0000) | 0.6000  (0.5333, 0.8667) | 0.4286  (0.3076, 0.6122) | 0.1332  (0.1062, 0.1613) |
| No MDT + Context | 0.7882  (0.6800, 0.8963) | 0.7895  (0.6206, 1.0000) | 0.8020  (0.6979, 0.8800) | 0.4286  (0.2469, 0.6001) | 0.9529  (0.8989, 1.0000) | 0.8000  (0.7000, 0.8750) | 0.5556  (0.3809, 0.7097) | 0.1198  (0.0881, 0.1520) |
| MDT + Context | 0.8280  (0.7378, 0.9183) | 0.8421  (0.6111, 1.0000) | 0.7426  (0.5427, 0.9375) | 0.3810  (0.2414, 0.7098) | 0.9615  (0.9121, 1.0000) | 0.7583  (0.6081, 0.9083) | 0.5246  (0.3823, 0.7420) | 0.1173  (0.0856, 0.1494) |

**Supplementary Table 7.** Mapping of the 15 predictors between JSON variable names and full clinical names selected via LASSO regression.

| **Variable name** | **Full clinical name** |
| --- | --- |
| age | Age |
| stroke | History of stroke |
| hemoglobin | Hemoglobin level |
| hdl | High-density lipoprotein cholesterol |
| preoperative fasting blood glucose | Preoperative fasting blood glucose level |
| preoperative myoglobin | Preoperative myoglobin level |
| preoperative limb ischemia | Preoperative limb ischemia |
| entry tear | Location of the primary entry tear |
| bovine arch | Bovine arch |
| ligation of sca without subsequent reconstruction | Ligation of the left subclavian artery without subsequent reconstruction |
| cpb time | Cardiopulmonary bypass time |
| cannulation site | Cannulation site |
| cerebral perfusion | Cerebral perfusion strategy |
| intraoperative platelets transfusion | Intraoperative platelet transfusion volume |
| max lactate intraoperation | Peak intraoperative lactate level |

**Supplementary Figure 1.** Repeated predicted probabilities and distribution of within-patient variability metrics across 5 independent GPT-5 runs under four application settings: (A) GPT-5 + No MDT + No Context; (B) GPT-5 + MDT + No Context; (C) GPT-5 + No MDT + Context; (D) GPT-5 + MDT + Context.

**Supplementary Figure 2.** Decision curve analysis and calibration plots of the LLM models: (A) Decision curve analysis of the four GPT-5-based prediction modes; (B) Calibration plots of the four GPT-5-based prediction modes; (C) Decision curve analysis of the four DeepSeek-V3-based prediction modes; (D) Calibration plots of the four DeepSeek-V3-based prediction modes.

**Supplementary Example A:** Output of a single-agent LLM prediction without in-context learning.

--------------------------------------------------------------------------------

System: You are a cardiovascular surgeon specializing in the treatment and management of patients with acute type A aortic dissection. You could assess the risk of postoperative neurological complications (PNC) using patients' basic information, key preoperative examination results, and intraoperative data. Now, please predict the risk (a probability value between 0 and 1) of postoperative neurological complications for the following patient and provide a brief rationale explaining the basis for your prediction.

The specific output format should be:

"Predicted probability of PNC: [your predicted probability]"

"Rationale for PNC prediction: [your prediction rationale]"

--------------------------------------------------------------------------------

User: Patient Profile: A 59-year-old male patient presented with acute Type A aortic dissection. The BMI was 28.5 kg/m². The patient reports smoking but denies alcohol use.

Past Medical History: Notable for hypertension. There is no history of arrhythmia, diabetes, Marfan syndrome, renal insufficiency, coronary artery disease, cerebrovascular disease, carotid atherosclerosis, thromboembolic disease, valvular heart disease, chronic obstructive pulmonary disease, thyroid disorder, or previous surgical procedures.

Preoperative Laboratory Findings:

• CBC: Hemoglobin 129.00 g/L; WBC 15.44 x 10⁹/L; Platelets 94.00 x 10⁹/L; Neutrophil Percentage 92.50 %; Absolute Neutrophil Count 14.28 x 10⁹/L; Lymphocyte count 0.39 x 10⁹/L; Monocyte count 0.76 x 10⁹/L.

• Coagulation: PT 12.60 s; INR 1.10; Fibrinogen 1.47 g/L; D-Dimer 38.00 mg/L FEU.

• Thyroid Function: TSH: 1.61 mIU/L; T3: 1.52 nmol/L; FT3: 4.49 pmol/L; T4: 106.00 nmol/L; FT4: 18.10 pmol/L.

• Chemistry and Cardiac Markers: Creatinine 158.00 μmol/L; Uric Acid 412.00 μmol/L; Albumin 44.30 g/L; Total Cholesterol 5.27 mmol/L; LDL 2.61 mmol/L; HDL 0.79 mmol/L; Triglycerides 5.03 mmol/L; Total Bilirubin 11.40 μmol/L; preoperative fasting blood glucose 12.82 mmol/L; ALT 18.00 IU/L; AST 17.00 IU/L; Myoglobin 58.73 ng/mL; BNP 1121.00 ng/L; CRP 6.94 mg/L.

Preoperative Imaging and Status:

• Blood Gas: Preoperative PaO₂ 58.30 mmHg; Preoperative PaCO₂ 53.20 mmHg.

• Anatomy & Status: The patient had no preoperative hypotension; no altered mental status; no limb ischemia; Entry tear was in the ascending aorta; no retrograde dissection; no bovine arch; The superior mesenteric artery originated from the true lumen; Moderate pericardial effusion was noted (≈101–299 ml).

• Procedure: total arch replacement with implantation of frozen elephant trunk; aortic root preserved; no coronary artery bypass grafting; The left subclavian artery was preserved or reconstructed.

• Parameters: Operation duration 7.70 h; CPB time 196.00 min; aortic cross-clamp time 144.00 min; lower body circulatory arrest time 31.00 min; lowest core temperature 23.00 °C.

• Perfusion and Transfusion: Cannulation via femoral artery; unilateral antegrade cerebral perfusion. Intraoperative transfusion included Fresh Frozen Plasma 0.00 ml; Packed Red Blood Cells 3.00 unit; platelets 2.00 unit; total intraoperative fluid 1850.00 ml; maximum intraoperative glucose 16.50 mmol/L; maximum intraoperative lactate 15.10 mmol/L.

--------------------------------------------------------------------------------

Assistant: "Predicted probability of PNC: 0.40"

"Rationale for PNC prediction: Elevated risk from femoral artery cannulation with total arch replacement + frozen elephant trunk (higher embolic risk) and unilateral antegrade cerebral perfusion; prolonged CPB (196 min), cross-clamp (144 min), and circulatory arrest (31 min); severe intraoperative hyperlactatemia (15.1 mmol/L) and marked perioperative hyperglycemia; preoperative hypoxemia/hypercapnia; coagulopathy and thrombocytopenia (platelets 94 x10⁹/L, fibrinogen 1.47 g/L, D-dimer 38 mg/L FEU); renal dysfunction (creatinine 158 μmol/L); smoking and hypertension. Mitigating factors include age 59, no preoperative hypotension/neurologic deficit, SMA true lumen, and deep hypothermia to 23°C."

**Supplementary Example B:** Output of a single-agent LLM prediction with in-context learning.

--------------------------------------------------------------------------------

System: You are a cardiovascular surgeon specializing in the treatment and management of patients with acute type A aortic dissection.

You will be provided with aggregated retrospective data from previous patients, including the entire cohort, a group that developed postoperative neurological complications (PNC), and a group that did not.

The variables cover basic patient information, key preoperative examination results, and intraoperative data.

For categorical variables, proportions within each group will be provided along with the P-value from chi-square tests (or Fisher's exact test, if applicable) comparing the PNC and non-PNC groups.

For continuous variables, if normally distributed, means and standard deviations will be provided, along with the P-value from t-tests; if non-normally distributed, medians and interquartile ranges (or quartiles) will be provided, along with the P-value from Mann-Whitney U tests.

Each variable will include data from all three groups as well as the P-value for the comparison of PNC and non-PNC groups.

Your task is to use these data to understand the characteristics of this patient population and the patterns of postoperative PNC occurrence, identify key risk factors potentially associated with PNC, and accumulate this knowledge as a foundation for subsequent tasks.

Here is the training data:

.....(Transmitted via json format, See Supplementary Data)

Based on the training data provided above, predict the risk (0-1 probability) of postoperative neurological complications (PNC) for this new patient. Important: In your rationale, describe risk factors in clinical terms without mentioning p-values or statistical significance.

Focus on clinically relevant associations.

The specific output format should be:

"Predicted probability of PNC: [your predicted probability]"

"Rationale for PNC prediction: [your prediction rationale]"

--------------------------------------------------------------------------------

User: Patient Profile: A 57-year-old male patient presented with acute Type A aortic dissection. The BMI was 21.9 kg/m². The patient reports smoking and alcohol use.

Past Medical History: Notable for chronic obstructive pulmonary disease. There is no history of hypertension, arrhythmia, diabetes, Marfan syndrome, renal insufficiency, coronary artery disease, cerebrovascular disease, carotid atherosclerosis, thromboembolic disease, valvular heart disease, thyroid disorder, or previous surgical procedures.

Preoperative Laboratory Findings:

• CBC: Hemoglobin 133.00 g/L; WBC 23.25 x 10⁹/L; Platelets 142.00 x 10⁹/L; Neutrophil Percentage 92.10 %; Absolute Neutrophil Count 21.41 x 10⁹/L; Lymphocyte count 0.81 x 10⁹/L; Monocyte count 1.00 x 10⁹/L.

• Coagulation: PT 11.70 s; INR 1.00; Fibrinogen 1.15 g/L; D-Dimer 38.00 mg/L FEU.

• Thyroid Function: TSH: 1.03 mIU/L; T3: 2.25 nmol/L; FT3: 5.40 pmol/L; T4: 115.00 nmol/L; FT4: 18.60 pmol/L.

• Chemistry and Cardiac Markers: Creatinine 71.00 μmol/L; Uric Acid 527.00 μmol/L; Albumin 43.10 g/L; Total Cholesterol 4.16 mmol/L; LDL 2.73 mmol/L; HDL 0.92 mmol/L; Triglycerides 2.02 mmol/L; Total Bilirubin 10.00 μmol/L; preoperative fasting blood glucose 7.25 mmol/L; ALT 14.00 IU/L; AST 37.00 IU/L; Myoglobin 3000.00 ng/mL; BNP 136.00 ng/L; CRP 3.01 mg/L.

Preoperative Imaging and Status:

• Blood Gas: Preoperative PaO₂ 99.00 mmHg; Preoperative PaCO₂ 48.60 mmHg.

• Anatomy & Status: The patient presented with preoperative hypotension; no altered mental status; limb ischemia present; Entry tear was in the aortic arch; no retrograde dissection; no bovine arch; The superior mesenteric artery was partially involved; Mild pericardial effusion was noted (≈50–100 ml).

• Procedure: total arch replacement with implantation of frozen elephant trunk; aortic root preserved; no coronary artery bypass grafting; The left subclavian artery was preserved or reconstructed.

• Parameters: Operation duration 7.50 h; CPB time 204.00 min; aortic cross-clamp time 102.00 min; lower body circulatory arrest time 23.00 min; lowest core temperature 24.00 °C.

• Perfusion and Transfusion: Cannulation via femoral artery; unilateral antegrade cerebral perfusion. Intraoperative transfusion included Fresh Frozen Plasma 350.00 ml; Packed Red Blood Cells 1.50 unit; platelets 1.00 unit; total intraoperative fluid 1500.00 ml; maximum intraoperative glucose 9.30 mmol/L; maximum intraoperative lactate 14.20 mmol/L.

--------------------------------------------------------------------------------

Assistant: Predicted probability of PNC: 0.44

Rationale for PNC prediction:

-Malperfusion and hypoperfusion burden: Preoperative limb ischemia and hypotension indicate systemic malperfusion; extremely high lactate (14.2 mmol/L) and markedly elevated myoglobin (~3000 ng/mL) reflect profound tissue ischemia, both associated with higher postoperative neurological injury risk.

-Inflammatory/stress profile: Very high WBC/neutrophilia with relative lymphopenia suggests severe stress/inflammation, which correlates with worse cerebral outcomes.

-Embolic risk related to perfusion strategy: Femoral (retrograde) cannulation can increase cerebral embolic load compared with axillary perfusion.

-Operative factors: Operation duration (7.5 h) and CPB time (204 min) are toward the higher-risk range for the cohort.

-Metabolic/coagulation milieu: Preoperative fasting glucose is elevated (7.25 mmol/L), and the combination of low fibrinogen (1.15 g/L) with very high D‑dimer suggests consumptive coagulopathy, increasing risks of microthrombosis or bleeding-related cerebral events.

-Pulmonary/ventilatory status: COPD with preoperative hypercapnia (PaCO2 48.6 mmHg) may reduce cerebral reserve during and after circulatory arrest and reperfusion.

-Factors tempering risk: Short cross-clamp time (102 min), preserved/reconstructed left subclavian artery (avoiding subclavian ligation), absence of bovine arch, relatively adequate platelet count, low BNP, modest transfusion volumes, and controlled intraoperative glucose.

On balance, multiple strong malperfusion and embolic-risk features outweigh the protective factors, yielding a substantially higher-than-baseline risk estimate.

**Supplementary Example C:** Transcript of a multi-agent discussion (MDT without in-context learning).

--------------------------------------------------------------------------------

Supervisor (to chat_manager):

As a medical expert team, analyze the following patient case to predict the risk of postoperative neurological complications (PNC) after acute type A aortic dissection surgery.

Provide your final prediction as a probability value between 0 and 1 with supporting rationale.

Patient Case:

Patient Profile: A 57-year-old male patient presented with acute Type A aortic dissection. The BMI was 21.9 kg/m². The patient reports smoking and alcohol use.

Past Medical History: Notable for chronic obstructive pulmonary disease. There is no history of hypertension, arrhythmia, diabetes, Marfan syndrome, renal insufficiency, coronary artery disease, cerebrovascular disease, carotid atherosclerosis, thromboembolic disease, valvular heart disease, thyroid disorder, or previous surgical procedures.

Preoperative Laboratory Findings:

• CBC: Hemoglobin 133.00 g/L; WBC 23.25 x 10⁹/L; Platelets 142.00 x 10⁹/L; Neutrophil Percentage 92.10 %; Absolute Neutrophil Count 21.41 x 10⁹/L; Lymphocyte count 0.81 x 10⁹/L; Monocyte count 1.00 x 10⁹/L.

• Coagulation: PT 11.70 s; INR 1.00; Fibrinogen 1.15 g/L; D-Dimer 38.00 mg/L FEU.

• Thyroid Function: TSH: 1.03 mIU/L; T3: 2.25 nmol/L; FT3: 5.40 pmol/L; T4: 115.00 nmol/L; FT4: 18.60 pmol/L.

• Chemistry and Cardiac Markers: Creatinine 71.00 µmol/L; Uric Acid 527.00 µmol/L; Albumin 43.10 g/L; Total Cholesterol 4.16 mmol/L; LDL 2.73 mmol/L; HDL 0.92 mmol/L; Triglycerides 2.02 mmol/L; Total Bilirubin 10.00 µmol/L; preoperative fasting blood glucose 7.25 mmol/L; ALT 14.00 IU/L; AST 37.00 IU/L; Myoglobin 3000.00 ng/mL; BNP 136.00 ng/L; CRP 3.01 mg/L.

Preoperative Imaging and Status:

• Blood Gas: Preoperative PaO₂99.00 mmHg; Preoperative PaCO₂48.60 mmHg.

• Anatomy & Status: The patient presented with preoperative hypotension; no altered mental status; limb ischemia present; Entry tear was in the aortic arch; no retrograde dissection; no bovine arch; The superior mesenteric artery was partially involved; Mild pericardial effusion was noted (~50–100 ml).

• Procedure: total arch replacement with implantation of frozen elephant trunk; aortic root preserved; no coronary artery bypass grafting; The left subclavian artery was preserved or reconstructed.

• Parameters: Operation duration 7.50 h; CPB time 204.00 min; aortic cross-clamp time 102.00 min; lower body circulatory arrest time 23.00 min; lowest core temperature 24.00 °C.

• Perfusion and Transfusion: Cannulation via femoral artery; unilateral antegrade cerebral perfusion. Intraoperative transfusion included Fresh Frozen Plasma 350.00 ml; Packed Red Blood Cells 1.50 unit; platelets 1.00 unit; total intraoperative fluid 1500.00 ml; maximum intraoperative glucose 9.30 mmol/L; maximum intraoperative lactate 14.20 mmol/L.

--------------------------------------------------------------------------------

Next speaker: Cardiovascular_Surgeon

Cardiovascular_Surgeon (to chat_manager):

Predicted probability of PNC: 0.307

Rationale for PNC prediction: Extensive arch pathology requiring total arch replacement with frozen elephant trunk increases embolic risk, amplified by initial femoral cannulation (retrograde aortic flow) before arch isolation; unilateral antegrade cerebral perfusion at moderate hypothermia (24°C) carries adequacy risk for territories reliant on the circle of Willis despite left subclavian preservation/reconstruction; CPB 204 min and cross-clamp 102 min lengthen inflammatory/embolic exposure; very high intraoperative lactate (14.2 mmol/L) signals significant global malperfusion during CPB, correlating with neurological injury; preoperative hypotension with limb and SMA malperfusion suggests unstable perfusion reserve and susceptibility to cerebral hypoperfusion intraoperatively; coagulopathy (fibrinogen 1.15 g/L, platelets 142 x10⁹/L) raises hemorrhagic/hemorrhagic-conversion risk. Protective factors include short lower-body circulatory arrest (23 min) under continuous ACP and absence of preoperative neurological deficit or carotid disease, tempering but not negating the above risks. This balance yields a moderate PNC risk estimated at 0.307.

--------------------------------------------------------------------------------

Next speaker: Anesthesiologist

Anesthesiologist (to chat_manager):

Predicted probability of PNC: 0.352

Rationale for PNC prediction: Anesthetic/intraoperative factors notably elevate risk: unilateral antegrade cerebral perfusion at a nadir 24°C during total arch replacement with initial femoral (retrograde) cannulation raises embolic and watershed hypoperfusion risk; prolonged CPB duration (204 min) increases inflammatory and microembolic burden; an extremely high peak lactate (14.2 mmol/L) signals severe global hypoperfusion during CPB and strongly correlates with neurological injury; mild intraoperative hyperglycemia (9.3 mmol/L) adds metabolic vulnerability; preoperative hypofibrinogenemia (1.15 g/L) with relatively limited intraoperative component replacement (FFP 350 ml, platelets 1 unit, PRBC 1.5 units) increases the likelihood of bleeding and hemorrhagic conversion. Protective elements include short lower-body circulatory arrest (23 min) under continuous ACP and preserved subclavian flow; net effect is a moderate-to-high PNC risk driven primarily by perfusion strategy, temperature level, prolonged CPB, and severe hyperlactatemia.

--------------------------------------------------------------------------------

Next speaker: Neurologist

Neurologist (to chat_manager):

Predicted probability of PNC: 0.331

Rationale for PNC prediction: Neurologically, baseline exam showed no altered mental status and there is no documented carotid/cerebrovascular disease, which is protective. However, several cerebrovascular risk markers are prominent: the dissection entry in the aortic arch places thrombus/debris adjacent to supra-aortic origins, increasing cerebral embolization risk; unilateral antegrade cerebral perfusion at 24°C relies on an intact circle of Willis, creating vulnerability of the contralateral hemisphere/posterior circulation in the absence of confirmed collateral adequacy; preoperative hypotension with limb/SMA malperfusion indicates systemic malperfusion that likely reduced cerebral perfusion reserve. The extremely high intraoperative lactate (14.2 mmol/L) is a strong surrogate of global hypoperfusion and correlates with postoperative stroke/encephalopathy. Coagulation profile (fibrinogen 1.15 g/L, platelets 142 x10⁹/L) increases the risk of hemorrhagic conversion if ischemic injury occurs. Additional neurologically relevant contributors include marked systemic inflammation (WBC 23.25 x10⁹/L, neutrophils 92.1%) predisposing to delirium and BBB dysfunction, smoking with low HDL suggesting reduced cerebrovascular reserve, mild hypercapnia (PaCO₂ 48.6 mmHg) implying altered cerebrovascular reactivity, and stress hyperglycemia (preop 7.25, intraop peak 9.3 mmol/L) which worsens ischemic brain vulnerability. Lack of preoperative deficits and absent known carotid disease modestly mitigate risk, but the cumulative cerebrovascular factors support a moderate PNC probability of 0.331.

--------------------------------------------------------------------------------

Next speaker: Supervisor

Supervisor (to chat_manager):

Extracted probabilities: Cardiovascular Surgeon: 0.307, Anesthesiologist: 0.352, Neurologist: 0.331

Predicted probability of PNC: 0.330

Rationale for PNC prediction: The team aligns on a moderate PNC risk driven by: total arch replacement with frozen elephant trunk and initial femoral (retrograde) cannulation increasing embolic load; unilateral antegrade cerebral perfusion at 24°C with unverified circle of Willis collaterals risking hemispheric/posterior circulation hypoperfusion; prolonged CPB (204 min) and cross-clamp (102 min) durations; and a markedly elevated peak lactate (14.2 mmol/L) signaling significant global hypoperfusion, strongly associated with postoperative neurological injury. Additional contributors include preoperative hypotension with limb/SMA malperfusion, coagulopathy (fibrinogen 1.15 g/L, platelets 142 x10⁹/L) increasing hemorrhagic conversion risk, systemic inflammation (WBC 23.25 x10⁹/L, neutrophils 92.1%), and perioperative hyperglycemia. Protective factors moderating risk include continuous ACP during a short lower-body circulatory arrest (23 min), preserved/reconstructed left subclavian flow, absence of preoperative neurologic deficits or known carotid disease, and preserved renal function. Key uncertainties noted by all specialists include lack of cerebral oximetry/ACP flow data and unconfirmed collateral adequacy; despite these gaps, the narrow range of estimates supports consensus at 0.330.

--------------------------------------------------------------------------------

>>>>>>>> TERMINATING RUN

**Supplementary Example D:** Transcript of a multi-agent discussion reaching the maximum 17 rounds (MDT with in-context learning).

--------------------------------------------------------------------------------

Supervisor (to chat_manager):

REFERENCE TRAINING DATA (for background knowledge only):

You will be provided with aggregated retrospective data from previous patients, including the entire cohort, a group that developed postoperative neurological complications (PNC), and a group that did not.

The variables cover basic patient information, key preoperative examination results, and intraoperative data.

For categorical variables, proportions within each group will be provided along with the P-value from chi-square tests (or Fisher's exact test, if applicable) comparing the PNC and non-PNC groups.

For continuous variables, if normally distributed, means and standard deviations will be provided, along with the P-value from t-tests; if non-normally distributed, medians and interquartile ranges (or quartiles) will be provided, along with the P-value from Mann-Whitney U tests.

Each variable will include data from all three groups as well as the P-value for the comparison of PNC and non-PNC groups.

Your task is to use these data to understand the characteristics of this patient population and the patterns of postoperative PNC occurrence, identify key risk factors potentially associated with PNC, and accumulate this knowledge as a foundation for subsequent tasks.

Here is the training data:

.....(Transmitted via json format, See Supplementary Data)

Please use this population-level data as reference when analyzing individual patient cases.

As a medical expert team, analyze the following new patient case to predict the risk of postoperative neurological complications (PNC) after acute aortic dissection surgery.

Provide your final prediction as a probability value between 0 and 1 with supporting rationale. Important: In your rationale, describe risk factors in clinical terms without mentioning p-values or statistical significance. Focus on clinically relevant associations.

Patient Case:

Patient Profile: A 59-year-old male patient presented with acute Type A aortic dissection. The BMI was 28.5 kg/m². The patient reports smoking but denies alcohol use.

Past Medical History: Notable for hypertension. There is no history of arrhythmia, diabetes, Marfan syndrome, renal insufficiency, coronary artery disease, cerebrovascular disease, carotid atherosclerosis, thromboembolic disease, valvular heart disease, chronic obstructive pulmonary disease, thyroid disorder, or previous surgical procedures.

Preoperative Laboratory Findings:

• CBC: Hemoglobin 129.00 g/L; WBC 15.44 x 10⁹/L; Platelets 94.00 x 10⁹/L; Neutrophil Percentage 92.50 %; Absolute Neutrophil Count 14.28 x 10⁹/L; Lymphocyte count 0.39 x 10⁹/L; Monocyte count 0.76 x 10⁹/L.

• Coagulation: PT 12.60 s; INR 1.10; Fibrinogen 1.47 g/L; D-Dimer 38.00 mg/L FEU.

• Thyroid Function: TSH: 1.61 mIU/L; T3: 1.52 nmol/L; FT3: 4.49 pmol/L; T4: 106.00 nmol/L; FT4: 18.10 pmol/L.

• Chemistry and Cardiac Markers: Creatinine 158.00 μmol/L; Uric Acid 412.00 μmol/L; Albumin 44.30 g/L; Total Cholesterol 5.27 mmol/L; LDL 2.61 mmol/L; HDL 0.79 mmol/L; Triglycerides 5.03 mmol/L; Total Bilirubin 11.40 μmol/L; preoperative fasting blood glucose 12.82 mmol/L; ALT 18.00 IU/L; AST 17.00 IU/L; Myoglobin 58.73 ng/mL; BNP 1121.00 ng/L; CRP 6.94 mg/L.

Preoperative Imaging and Status:

• Blood Gas: Preoperative PaO2 58.30 mmHg; Preoperative PaCO2 53.20 mmHg.

• Anatomy & Status: The patient had no preoperative hypotension; no altered mental status; no limb ischemia; Entry tear was in the ascending aorta; no retrograde dissection; no bovine arch; The superior mesenteric artery originated from the true lumen; Moderate pericardial effusion was noted (≈101–299 ml).

• Procedure: total arch replacement with implantation of frozen elephant trunk; aortic root preserved; no coronary artery bypass grafting; The left subclavian artery was preserved or reconstructed.

• Parameters: Operation duration 7.70 h; CPB time 196.00 min; aortic cross-clamp time 144.00 min; lower body circulatory arrest time 31.00 min; lowest core temperature 23.00°C.

• Perfusion and Transfusion: Cannulation via femoral artery; unilateral antegrade cerebral perfusion. Intraoperative transfusion included Fresh Frozen Plasma 0.00 ml; Packed Red Blood Cells 3.00 unit; platelets 2.00 unit; total intraoperative fluid 1850.00 ml; maximum intraoperative glucose 16.50 mmol/L; maximum intraoperative lactate 15.10 mmol/L.

--------------------------------------------------------------------------------

Next speaker: Cardiovascular_Surgeon

Cardiovascular_Surgeon (to chat_manager):

Predicted probability of PNC: 0.376

Rationale for PNC prediction:

- Arch complexity and embolic risk: Total arch replacement with frozen elephant trunk entails extensive arch manipulation, which increases embolic load to the brain. In cohort patterns, more complex arch work aligns with higher neurological risk, even though arch type alone was not decisive.

- Cannulation strategy: Femoral (arteria cruralis) cannulation has been associated with higher postoperative neurological complications compared with axillary access, likely from retrograde athero/thromboembolism into the arch and cerebral vessels. This patient’s femoral cannulation is an adverse factor.

- Cerebral perfusion approach: Unilateral antegrade cerebral perfusion is acceptable but provides less margin than bilateral perfusion if the circle of Willis is incomplete. Compared with retrograde perfusion it is favorable, but relative to bilateral strategies it confers a modestly higher risk.

- Circulatory arrest and temperature: Lower body circulatory arrest time of 31 minutes at a nadir temperature of 23°C is within a protective window for cerebral ischemia, which moderates risk.

- Procedural duration and bypass metrics: Operation time (7.7 h) is on the higher side of the cohort and correlates with increased neurological complications; CPB time (196 min) and cross-clamp time (144 min) are mid-range but still contribute to cumulative embolic and inflammatory burden.

- Intraoperative physiology: Markedly elevated peak lactate (15.1 mmol/L) indicates significant global hypoperfusion/malperfusion during the case and is strongly associated with higher postoperative neurological injury in the cohort. Marked intraoperative hyperglycemia (16.5 mmol/L) also tracks with worse neurological outcomes.

- Transfusion profile: Platelet transfusion (2 units) aligns with cohort patterns where higher platelet transfusion accompanies greater neurological complications, reflecting coagulopathy and platelet dysfunction during complex arch surgery.

- Protective elements: Left subclavian artery preserved/reconstructed (avoids posterior circulation hypoperfusion seen when ligated), absence of bovine arch, and no preoperative neurologic deficit lower baseline risk.

Balancing these, the combination of femoral cannulation, unilateral cerebral perfusion, longer operation, and especially very high intraoperative lactate and hyperglycemia raises this patient’s risk above the cohort baseline, while controlled arrest time, deep hypothermia, LSA preservation, and mid-range CPB/ACC temper it. Hence an independent, surgery-focused estimate of 0.376.

--------------------------------------------------------------------------------

Next speaker: Anesthesiologist

Anesthesiologist (to chat_manager):

Predicted probability of PNC: 0.542

Rationale for PNC prediction:

- Global perfusion/metabolic stress: The peak intraoperative lactate was extremely high at 15.1 mmol/L, indicating significant hypoperfusion or low-flow episodes. In the reference cohort, higher intraoperative lactate strongly tracked with postoperative neurological complications; this patient’s value is far above typical ranges, making it a dominant risk factor.

- Hyperglycemia: Peak intraoperative glucose reached 16.5 mmol/L. Cohort patterns showed higher intraoperative glucose in those with PNC, and broader anesthetic literature links intraoperative hyperglycemia with worse neurologic outcomes, reinforcing elevated risk.

- Cerebral perfusion strategy: Unilateral antegrade cerebral perfusion provides less redundancy than bilateral strategies if the circle of Willis is incomplete. While more favorable than retrograde perfusion, it confers a modestly higher neurologic risk than bilateral perfusion in population patterns.

- Cannulation site: Femoral (arteria cruralis) cannulation was more common among PNC cases in the cohort, consistent with concern for retrograde embolization to cerebral vessels during arch work.

- Bypass and operative duration: Operation time (7.7 h) is on the higher side, and CPB time (196 min) is toward the longer end of cohort distributions associated with PNC, reflecting greater inflammatory and embolic burden.

- Temperature and arrest: Nadir temperature of 23°C with lower body circulatory arrest time of 31 minutes falls within commonly protective windows for cerebral ischemia, which moderates but does not offset the above risks.

- Coagulation/transfusion: Platelet transfusion of 2 units aligns with the cohort pattern where higher platelet transfusion was associated with PNC, likely reflecting coagulopathy/platelet dysfunction during complex arch surgery. PRBC and plasma amounts were not strongly discriminative in the cohort.

Taken together, the very high lactate and substantial hyperglycemia during CPB, combined with femoral cannulation, unilateral cerebral perfusion, and longer operative exposure, push the risk well above baseline despite acceptable hypothermia and arrest times.

--------------------------------------------------------------------------------

Next speaker: Neurologist

Neurologist (to chat_manager):

Predicted probability of PNC: 0.611

Rationale for PNC prediction:

- Baseline neurovascular vulnerability: Age 59 places the patient in a group with higher neurological complication rates in population data. Although there is no prior stroke, carotid disease, or preoperative neurological deficit, severe preoperative hypoxemia (PaO2 58 mmHg) with hypercapnia (PaCO2 53 mmHg) suggests compromised cerebral oxygenation reserve before surgery.

- Thrombo-hemorrhagic milieu unfavorable for the brain: Marked thrombocytopenia (platelets 94 x10⁹/L) with low fibrinogen (1.47 g/L) and very high D-dimer (38 mg/L FEU) indicates consumptive coagulopathy and an unstable hemostatic state. In cohort patterns, lower platelet counts tracked with PNC, consistent with increased risk of both ischemic embolic events and hemorrhagic complications in the brain.

- Systemic inflammatory/immune profile linked to encephalopathy risk: Profound lymphopenia (0.39 x10⁹/L) aligns with cohort observations of lower lymphocyte counts among PNC cases and is associated clinically with delirium/encephalopathy susceptibility after major surgery.

- Metabolic and end-organ factors that exacerbate cerebral injury: Preoperative hyperglycemia (fasting glucose 12.82 mmol/L) is associated with PNC in the cohort and is known to amplify ischemic neuronal injury. Elevated creatinine (158 μmol/L) reflects renal dysfunction, which correlates with postoperative encephalopathy risk. Elevated BNP indicates hemodynamic strain, which often coexists with impaired cerebral perfusion.

- Intraoperative cerebrovascular exposures: Femoral arterial cannulation increases retrograde cerebral microembolization risk compared with axillary access in the cohort. Unilateral antegrade cerebral perfusion offers less redundancy if the circle of Willis is incomplete. Prolonged operative exposure (7.7 h) and CPB duration near the higher range add embolic/inflammatory burden to the brain in population patterns.

- Strong hypoperfusion signal: The very high peak intraoperative lactate (15.1 mmol/L) indicates significant global low-flow states; in the cohort, higher lactate closely tracked with neurological complications. Severe intraoperative hyperglycemia (16.5 mmol/L) and intraoperative platelet transfusion (2 units) further align with the PNC profile.

- Moderating elements: Deep hypothermia (23°C) with a lower-body circulatory arrest time of 31 minutes and preservation/reconstruction of the left subclavian artery are protective; absence of a bovine arch and no preoperative neurological impairment also lower baseline risk. However, these are outweighed by the adverse neuro-perfusion profile and metabolic-hypoperfusion signals.

Integrating these neurologically focused factors with cohort patterns, the balance of risks supports a high probability of postoperative neurological complications.

--------------------------------------------------------------------------------

Next speaker: Supervisor

Supervisor (to chat_manager):

Extracted probabilities: Cardiovascular Surgeon: 0.376, Anesthesiologist: 0.542, Neurologist: 0.611

Consensus calculation:

- Average (simple): 0.510

- Range: 0.235 → Consensus not reached (range > 0.100)

Predicted probability of PNC: 0.510

Rationale for PNC prediction:

- Adverse intraoperative physiology: Extremely high peak lactate (15.1 mmol/L) indicates substantial hypoperfusion/low-flow exposure; severe intraoperative hyperglycemia (16.5 mmol/L) further exacerbates neuronal injury risk.

- Cannulation and cerebral perfusion: Femoral cannulation carries higher embolic risk than axillary approaches; unilateral antegrade cerebral perfusion provides less redundancy if the circle of Willis is incomplete.

- Procedural burden: Longer operation (7.7 h) and CPB duration (196 min) increase embolic/inflammatory load.

- Hemostatic/inflammatory profile: Preoperative thrombocytopenia (94 x10⁹/L) and lymphopenia (0.39 x10⁹/L) align with higher PNC risk patterns; low fibrinogen and very high D-dimer suggest a consumptive coagulopathy milieu unfavorable for cerebral outcomes.

- Metabolic/organ dysfunction: Markedly elevated preoperative fasting glucose (12.82 mmol/L) and renal dysfunction (creatinine 158 μmol/L) are linked to postoperative encephalopathy and stroke risk; high BNP reflects hemodynamic strain.

- Preoperative gas exchange: Hypoxemia (PaO2 58 mmHg) with hypercapnia (PaCO2 53 mmHg) reduces cerebral oxygenation reserve pre-surgery.

- Protective factors: Deep hypothermia (23°C) with lower-body arrest 31 minutes is within protective ranges; left subclavian artery preserved/reconstructed; no bovine arch; no preoperative limb ischemia or neurological deficit.

Note on training data use: All specialists referenced population-level patterns (e.g., effects of femoral cannulation, lower platelets/lymphocytes, higher pre/intraoperative glucose, longer CPB/operation times, platelet transfusion, and elevated intraoperative lactate) in forming their assessments.

--------------------------------------------------------------------------------

Next speaker: Cardiovascular_Surgeon

Cardiovascular_Surgeon (to chat_manager):

Predicted probability of PNC: 0.468

Rationale for PNC prediction:

- Arch complexity and embolic exposure: Total arch replacement with frozen elephant trunk entails extensive arch manipulation and stent deployment within the arch/descending thoracic aorta, increasing particulate and atherothrombotic embolic load to cerebral vessels in population patterns.

- Cannulation strategy: Femoral (arteria cruralis) cannulation is linked with higher neurologic events than axillary cannulation due to retrograde flow across the diseased arch, aligning with cohort patterns where femoral access was more common among PNC cases.

- Cerebral perfusion approach: Unilateral antegrade cerebral perfusion provides less redundancy than bilateral perfusion if the circle of Willis is incomplete, carrying a modestly higher neurological risk compared with bilateral strategies in the training cohort.

- Procedural and bypass burden: Operation time of 7.7 hours and CPB time of 196 minutes fall into ranges associated with more postoperative neurologic complications in the cohort, reflecting greater embolic and inflammatory exposure; aortic cross-clamp time (144 minutes) adds to this cumulative risk.

- Intraoperative physiology: Very high peak lactate (15.1 mmol/L) indicates significant low-flow/hypoperfusion exposure and strongly tracks with PNC in the cohort. Marked intraoperative hyperglycemia (16.5 mmol/L) similarly aligns with worse neurological outcomes.

- Transfusion/coagulopathy signal: Receipt of platelet transfusion (2 units) mirrors cohort patterns where greater platelet transfusion accompanied higher PNC rates, consistent with coagulopathy and platelet dysfunction during complex arch surgery.

- Moderating elements: Lower-body circulatory arrest time of 31 minutes at 23°C is within commonly protective windows for cerebral ischemia; preservation/reconstruction of the left subclavian artery avoids posterior circulation hypoperfusion seen when ligated; absence of a bovine arch and no need for subclavian ligation reduce risk.

Balancing the high-risk surgical exposures (femoral cannulation, unilateral cerebral perfusion, extensive arch/FET work, longer CPB/operative times, and especially very high intraoperative lactate with severe hyperglycemia) against protective arrest/temperature and LSA preservation, the surgery-focused probability is moderately high at 0.468.

--------------------------------------------------------------------------------

Next speaker: Anesthesiologist

Anesthesiologist (to chat_manager):

Predicted probability of PNC: 0.578

Rationale for PNC prediction:

- Perfusion/metabolic stress signals: The peak intraoperative lactate of 15.1 mmol/L is markedly elevated, indicating significant low-flow/hypoperfusion exposure during CPB; in the reference cohort, higher intraoperative lactate was strongly associated with PNC. The peak intraoperative glucose of 16.5 mmol/L is well above cohort medians and aligns with worse neurological outcomes in population patterns.

- Cardiopulmonary bypass and operative exposure: CPB time of 196 minutes and total operative time of 7.7 hours are in ranges associated with increased neurologic complications in the cohort, reflecting cumulative embolic and inflammatory burden.

- Cannulation and cerebral perfusion strategy: Femoral (arteria cruralis) cannulation was more frequent among PNC cases in the cohort, consistent with retrograde embolic risk during arch work. Unilateral antegrade cerebral perfusion, while acceptable, offers less redundancy than bilateral strategies and carries a modestly higher risk if collateral pathways are inadequate.

- Temperature and circulatory arrest: Deep hypothermia with a nadir of 23°C and lower-body circulatory arrest of 31 minutes fall within protective windows for cerebral ischemia, providing some mitigation but not offsetting the marked hypoperfusion and metabolic derangement signals.

- Coagulation/transfusion profile: Intraoperative platelet transfusion of 2 units mirrors the cohort pattern where greater platelet transfusion tracked with PNC, suggesting intraoperative coagulopathy/platelet dysfunction during complex arch surgery. PRBC and plasma volumes were not discriminative in the cohort.

Integrating these anesthesia- and intraoperative-specific factors with cohort patterns, the combination of very high lactate, severe intraoperative hyperglycemia, femoral cannulation, longer CPB/operative duration, and platelet transfusion places this patient’s risk well above baseline despite acceptable hypothermia and arrest parameters.

--------------------------------------------------------------------------------

Next speaker: Neurologist

Neurologist (to chat_manager):

Predicted probability of PNC: 0.642

Rationale for PNC prediction:

- Baseline cerebrovascular reserve is limited: age 59 and marked preoperative hypoxemia (PaO2 58 mmHg) with hypercapnia (PaCO2 53 mmHg) reduce cerebral oxygenation margin despite no prior stroke or carotid disease.

- Prothrombotic–coagulopathic profile unfavorable for the brain: thrombocytopenia (94 x10⁹/L), low fibrinogen (1.47 g/L), and very high D‑dimer (38 mg/L FEU) indicate consumptive coagulopathy, aligning with cohort patterns where lower platelets tracked with PNC and raising both ischemic and hemorrhagic cerebral risk.

- Immune/inflammatory signal: profound lymphopenia (0.39 x10⁹/L) parallels cohort trends in PNC and correlates clinically with postoperative encephalopathy/delirium vulnerability.

- Metabolic and end‑organ stressors that amplify neuronal injury: markedly elevated preoperative fasting glucose (12.82 mmol/L) and renal dysfunction (creatinine 158 μmol/L) are associated with higher rates of postoperative encephalopathy and stroke; elevated BNP reflects hemodynamic strain. Preoperative myoglobin is above typical non-PNC levels in the cohort, indicating systemic injury burden linked with worse neurologic outcomes.

- Intraoperative cerebrovascular exposures: femoral cannulation (retrograde embolic risk) and unilateral antegrade cerebral perfusion (less redundancy if the circle of Willis is incomplete) increase embolic/ischemic risk; longer CPB (196 min) and operative time (7.7 h) add embolic and inflammatory load.

- Strong hypoperfusion/metabolic insult: extremely high peak intraoperative lactate (15.1 mmol/L) and severe hyperglycemia (16.5 mmol/L) closely align with PNC patterns and suggest significant global low-flow episodes; platelet transfusion (2 units) further reflects intraoperative coagulopathy associated with neurologic complications in the cohort.

- Moderators: deep hypothermia at 23°C with 31 minutes of lower-body circulatory arrest, preserved/reconstructed left subclavian artery, absence of bovine arch, and no preoperative neurologic deficit provide partial mitigation but do not offset the above high-risk neurovascular signals.

Taken together, the cerebrovascular reserve limitations, coagulopathy, metabolic/renal risk, and strong intraoperative hypoperfusion markers support a high neurologically focused probability of PNC.

--------------------------------------------------------------------------------

Next speaker: Supervisor

Supervisor (to chat_manager):

Extracted probabilities: Cardiovascular Surgeon: 0.468, Anesthesiologist: 0.578, Neurologist: 0.642

Consensus calculation:

- Average (simple): 0.563

- Range: 0.174 → Consensus not reached (range > 0.100)

Predicted probability of PNC: 0.563

Rationale for PNC prediction:

- Marked intraoperative hypoperfusion/metabolic stress: peak lactate 15.1 mmol/L and hyperglycemia 16.5 mmol/L strongly align with higher postoperative neurological complications.

- Cannulation/perfusion strategy: femoral cannulation carries higher retrograde embolic risk; unilateral antegrade cerebral perfusion offers less redundancy if collateral flow is limited.

- Procedural burden: longer operation (7.7 h) and CPB duration (196 min) increase embolic and inflammatory exposure to the brain.

- Hemostatic/immune milieu: thrombocytopenia (94 x10⁹/L), low fibrinogen, very high D‑dimer, and profound lymphopenia are associated with ischemic and hemorrhagic brain injury and postoperative encephalopathy.

- Metabolic/organ dysfunction: preoperative hyperglycemia (12.82 mmol/L) and renal dysfunction (creatinine 158 μmol/L) heighten vulnerability to neurological complications.

- Preoperative gas exchange compromise: hypoxemia (PaO2 58 mmHg) with hypercapnia (PaCO2 53 mmHg) suggests reduced cerebral oxygen reserve.

- Moderators: deep hypothermia (23°C) with 31 minutes of lower-body arrest, preserved/reconstructed left subclavian artery, and absence of bovine arch provide partial protection but do not offset the above risks.

Training data use: All specialists incorporated cohort-level patterns (e.g., higher intraoperative lactate and glucose, femoral cannulation, lower platelets/lymphocytes, longer CPB/operative times, platelet transfusion) in forming their assessments.

--------------------------------------------------------------------------------

Next speaker: Cardiovascular_Surgeon

Cardiovascular_Surgeon (to chat_manager):

Predicted probability of PNC: 0.524

Rationale for PNC prediction:

- Arch complexity and embolic load: Total arch replacement with frozen elephant trunk requires extensive arch manipulation and endograft deployment, which increases cerebral particulate/atherothrombotic embolization risk. In cohort patterns, greater operative complexity and arch instrumentation aligned with higher neurological events.

- Cannulation strategy: Femoral cannulation exposes the arch and supra‑aortic vessels to retrograde flow across dissected/atheromatous surfaces. In the cohort, PNC was more frequent with femoral (and “other”) cannulation, whereas axillary access was less represented among PNC cases.

- Cerebral perfusion: Unilateral antegrade cerebral perfusion offers less redundancy than bilateral if collateral pathways are incomplete, carrying a modestly higher neurologic risk than bilateral strategies; it is preferable to retrograde perfusion but not optimal for uniform hemispheric protection in extensive arch work.

- Bypass and operative exposure: CPB time of 196 minutes and total operative time of 7.7 hours fall in ranges associated with more PNC in the cohort, reflecting cumulative embolic and inflammatory burden; the aortic cross‑clamp time (144 minutes) contributes additional risk.

- Intraoperative physiology: Peak lactate of 15.1 mmol/L strongly signals significant low‑flow/hypoperfusion exposure and closely tracked with PNC in the cohort. Marked intraoperative hyperglycemia (16.5 mmol/L) further aligns with worse neurologic outcomes.

- Transfusion/coagulopathy signal: Intraoperative platelet transfusion (2 units) mirrors the cohort pattern in which higher platelet use accompanied PNC, suggesting coagulopathy/platelet dysfunction during complex arch surgery.

- Protective elements tempering risk: Lower‑body circulatory arrest time of 31 minutes at 23°C is within commonly protective cerebral ischemia windows; preservation/reconstruction of the left subclavian artery avoids posterior circulation compromise, and absence of a bovine arch removes an anatomic risk signal seen in the cohort.

Weighing the adverse surgical exposures (femoral cannulation, unilateral ACP, extensive TAR+FET manipulation, prolonged CPB/operative times, and especially the very high intraoperative lactate with hyperglycemia) against the protective cooling/arrest parameters and LSA preservation yields a moderately high, surgery‑focused probability of 0.524.

--------------------------------------------------------------------------------

Next speaker: Anesthesiologist

Anesthesiologist (to chat_manager):

Predicted probability of PNC: 0.627

Rationale for PNC prediction: Intraoperative metabolic stress was profound: peak lactate 15.1 mmol/L and glucose 16.5 mmol/L indicate significant low‑flow/hypoperfusion and stress hyperglycemia, which in the cohort aligned strongly with postoperative neurologic complications. CPB duration (196 min) and total operative time (7.7 h) fall in ranges associated with greater embolic/inflammatory burden. Femoral cannulation adds retrograde embolic risk, and unilateral antegrade cerebral perfusion provides less redundancy than bilateral strategies, both tracking with higher neurologic risk in population patterns. Coagulation/transfusion signals (baseline thrombocytopenia with need for 2 units of platelets intraoperatively) mirror cohort profiles linked to PNC, suggesting intraoperative coagulopathy/platelet dysfunction. Temperature management and arrest parameters (23°C nadir, 31 min lower‑body circulatory arrest) are within protective ranges and temper risk but do not offset the marked hypoperfusion and metabolic derangement. Integrating these anesthesia- and perfusion-specific factors with training data patterns supports a high, above-baseline probability.

--------------------------------------------------------------------------------

Next speaker: Neurologist

Neurologist (to chat_manager):

Predicted probability of PNC: 0.671

Rationale for PNC prediction:

- Limited cerebrovascular reserve preoperatively: age 59 and substantial gas-exchange derangement (hypoxemia PaO2 58 mmHg with hypercapnia PaCO2 53 mmHg) reduce cerebral oxygenation margin despite no prior stroke or carotid disease, increasing susceptibility to perioperative encephalopathy and ischemic injury.

- Unfavorable hemostatic milieu: thrombocytopenia (94 x10⁹/L), low fibrinogen (1.47 g/L), and markedly elevated D‑dimer (38 mg/L FEU) indicate consumptive coagulopathy. In cohort patterns, lower platelets correlated with neurological complications; this profile raises risks of both ischemic stroke and intracranial hemorrhage/hemorrhagic transformation.

- Immune/inflammatory profile linked to postoperative brain dysfunction: profound lymphopenia (0.39 x10⁹/L) mirrored cohort trends among PNC patients and clinically aligns with vulnerability to postoperative delirium/encephalopathy.

- Metabolic and end-organ vulnerability that amplifies neuronal injury: very high preoperative fasting glucose (12.82 mmol/L) tracked with PNC in the cohort and worsens ischemic brain injury. Elevated creatinine (158 μmol/L) indicates renal dysfunction, which is associated with postoperative encephalopathy. Elevated BNP and above-typical myoglobin signify systemic/hemodynamic stress linked with worse neurological outcomes.

- Intraoperative cerebrovascular exposures: femoral cannulation increases retrograde cerebral microembolization risk; unilateral antegrade cerebral perfusion provides less collateral redundancy if the circle of Willis is incomplete. Longer CPB (196 min) and operative time (7.7 h) add embolic and inflammatory burden to the brain in population patterns.

- Strong hypoperfusion/metabolic insult: extremely high peak intraoperative lactate (15.1 mmol/L) and severe intraoperative hyperglycemia (16.5 mmol/L) strongly align with postoperative neurological complications, signaling significant global low-flow episodes. Platelet transfusion (2 units) further reflects intraoperative coagulopathy associated with neurologic injury profiles in the cohort.

- Moderating elements: deep hypothermia (23°C) with 31 minutes of lower-body circulatory arrest, preservation/reconstruction of the left subclavian artery, absence of a bovine arch, and no preoperative neurological deficit offer partial protection but do not counterbalance the dominant adverse neuro-perfusion and coagulopathy signals.

Integrating these neurologically focused factors with cohort patterns supports a high probability of postoperative neurological complications.

--------------------------------------------------------------------------------

Next speaker: Supervisor

Supervisor (to chat_manager):

Extracted probabilities: Cardiovascular Surgeon: 0.524, Anesthesiologist: 0.627, Neurologist: 0.671

Consensus calculation:

- Average (simple): 0.607

- Range: 0.147 → Consensus not reached (range > 0.100)

Predicted probability of PNC: 0.607

Rationale for PNC prediction:

- Dominant intraoperative risk signals: Extremely high peak lactate (15.1 mmol/L) and severe hyperglycemia (16.5 mmol/L) indicate significant low-flow/hypoperfusion and metabolic stress, which align with higher postoperative neurological complications in cohort data.

- Embolic/ischemic exposure: Femoral (retrograde) cannulation increases cerebral microembolization risk versus axillary access; unilateral antegrade cerebral perfusion offers less redundancy if collateral pathways are limited.

- Procedural burden: Prolonged operation (7.7 h) and CPB duration (196 min) increase embolic and inflammatory load to the brain.

- Preoperative vulnerability: Thrombocytopenia (94 x10⁹/L), low fibrinogen, very high D‑dimer, and profound lymphopenia indicate a coagulopathic/immune milieu linked to both ischemic and hemorrhagic brain injury; marked preoperative hyperglycemia (12.82 mmol/L), renal dysfunction (creatinine 158 μmol/L), and gas-exchange derangements (PaO2 58 mmHg, PaCO2 53 mmHg) further reduce neurologic resilience.

- Moderating factors: Deep hypothermia (23°C) with 31 minutes of lower-body circulatory arrest, preserved/reconstructed left subclavian artery, and absence of a bovine arch provide partial protection but do not offset the dominant adverse signals.

Training data use:

- The team explicitly referenced population-level patterns from the cohort (e.g., higher intraoperative lactate and glucose, femoral cannulation vs axillary, lower platelets/lymphocytes, longer CPB/operative times, and platelet transfusion) when forming their assessments.

--------------------------------------------------------------------------------

Next speaker: Cardiovascular_Surgeon

Cardiovascular_Surgeon (to chat_manager):

Predicted probability of PNC: 0.589

Rationale for PNC prediction:

- Extensive arch manipulation: Total arch replacement with frozen elephant trunk increases cerebral embolic exposure from arch instrumentation and stent deployment, raising neurological risk compared with limited arch work in cohort patterns.

- Cannulation strategy: Femoral (retrograde) cannulation is associated with higher neurologic complications than axillary access due to retrograde flow across diseased arch surfaces and greater embolic potential.

- Cerebral perfusion approach: Unilateral antegrade cerebral perfusion offers less hemispheric redundancy than bilateral strategies during complex arch reconstruction, adding modest ischemic risk if collateral pathways are inadequate.

- Procedural/bypass burden: Operation time 7.7 hours and CPB time 196 minutes fall within ranges linked to higher neurological complications in the cohort, reflecting cumulative embolic and inflammatory load; cross-clamp time of 144 minutes contributes to this exposure.

- Arrest and temperature: Lower-body circulatory arrest of 31 minutes at 23°C is within commonly protective cerebral windows, tempering risk but not negating other adverse factors.

- Intraoperative perfusion adequacy signals: Very high peak lactate (15.1 mmol/L) and marked intraoperative hyperglycemia (16.5 mmol/L) indicate significant low-flow/hypoperfusion and metabolic stress during CPB; in cohort data, higher lactate strongly tracked with postoperative neurological complications.

- Coagulopathy/transfusion marker: Intraoperative platelet transfusion (2 units) aligns with cohort patterns linking greater platelet use with PNC in complex arch surgery, suggesting intraoperative coagulopathy/platelet dysfunction.

- Protective elements: Left subclavian artery preserved/reconstructed (avoiding posterior circulation compromise seen with ligation), absence of bovine arch, and use of antegrade (rather than retrograde) cerebral perfusion provide partial mitigation.

Integrating these surgery-specific exposures with cohort patterns, the combination of femoral cannulation, unilateral cerebral perfusion, extensive TAR+FET manipulation, prolonged CPB/operative times, and especially the marked intraoperative lactate signal supports a moderately high PNC risk estimate.

--------------------------------------------------------------------------------

Next speaker: Anesthesiologist

Anesthesiologist (to chat_manager):

Predicted probability of PNC: 0.646

Rationale for PNC prediction:

- Perfusion/metabolic stress: The peak intraoperative lactate of 15.1 mmol/L is markedly above typical cohort values and strongly aligns with postoperative neurological complications, signaling significant low‑flow/hypoperfusion during CPB. The peak intraoperative glucose of 16.5 mmol/L further indicates stress hyperglycemia associated with worse neurologic outcomes.

- Bypass and operative exposure: CPB time of 196 minutes and total operative time of 7.7 hours fall in ranges associated with higher neurological complications in the cohort, reflecting cumulative embolic and inflammatory burden to the brain.

- Cannulation and cerebral perfusion: Femoral (retrograde) cannulation carries higher cerebral microembolization risk versus axillary access in population patterns. Unilateral antegrade cerebral perfusion provides less collateral redundancy than bilateral strategies, adding ischemic vulnerability if the circle of Willis is incomplete.

- Temperature/arrest: Deep hypothermia to 23°C and lower‑body circulatory arrest of 31 minutes are within protective windows for cerebral ischemia, which moderates but does not counterbalance the strong hypoperfusion and metabolic risk signals.

- Coagulation/transfusion indicators: Intraoperative platelet transfusion of 2 units mirrors cohort profiles where greater platelet use tracked with PNC, suggesting intraoperative coagulopathy/platelet dysfunction during complex arch reconstruction. PRBC and plasma amounts were not discriminative in the cohort.

Integrating these anesthesia- and intraoperative-specific factors with cohort patterns supports a high probability of postoperative neurological complications.

--------------------------------------------------------------------------------

Next speaker: Neurologist

Neurologist (to chat_manager):

Predicted probability of PNC: 0.692

Rationale for PNC prediction: From a neurologic standpoint, this patient has limited cerebrovascular reserve preoperatively (age 59 with marked hypoxemia PaO2 58 mmHg and hypercapnia PaCO2 53 mmHg), an unfavorable hemostatic milieu (platelets 94 x10⁹/L, fibrinogen 1.47 g/L, D‑dimer 38 mg/L FEU) and immune profile (lymphocytes 0.39 x10⁹/L), and metabolic/organ vulnerabilities that amplify cerebral injury (fasting glucose 12.82 mmol/L, creatinine 158 μmol/L, elevated BNP), all of which align with higher postoperative neurological complications in cohort patterns; intraoperatively, femoral cannulation and unilateral antegrade cerebral perfusion increase embolic/ischemic exposure, prolonged CPB/operative times add inflammatory and microembolic burden, and the extremely high peak lactate (15.1 mmol/L) with severe hyperglycemia (16.5 mmol/L) signal significant global low‑flow episodes strongly linked to neurologic injury, while platelet transfusion (2 units) further reflects coagulopathy associated with PNC; although deep hypothermia with 31 minutes of arrest, preserved/reconstructed left subclavian artery, absence of a bovine arch, and no preoperative neurologic deficit offer partial mitigation, the aggregate neurovascular risk profile supports a high probability of postoperative neurological complications.

--------------------------------------------------------------------------------

Next speaker: Supervisor

Supervisor (to chat_manager):

Extracted probabilities: Cardiovascular Surgeon: 0.589, Anesthesiologist: 0.646, Neurologist: 0.692

Consensus calculation:

- Average (simple): 0.642

- Range: 0.103 → Consensus not reached (range > 0.100)

Predicted probability of PNC: 0.642

Rationale for PNC prediction:

- Dominant intraoperative stress signals: Extremely high peak lactate (15.1 mmol/L) and severe hyperglycemia (16.5 mmol/L) indicate significant low-flow/hypoperfusion and metabolic stress, which track with higher postoperative neurological complications in cohort patterns.

- Embolic/ischemic exposure: Femoral (retrograde) cannulation increases cerebral microembolization risk versus axillary access; unilateral antegrade cerebral perfusion provides less redundancy if collateral pathways are incomplete.

- Procedural burden: Prolonged operation (7.7 h) and CPB duration (196 min) add embolic and inflammatory load to the brain.

- Preoperative vulnerability: Hypoxemia (PaO2 58 mmHg) and hypercapnia (PaCO2 53 mmHg) suggest limited cerebrovascular reserve; thrombocytopenia (94 x10⁹/L), low fibrinogen, very high D‑dimer, and profound lymphopenia indicate a coagulopathic/immune milieu unfavorable for cerebral outcomes; fasting hyperglycemia (12.82 mmol/L), renal dysfunction (creatinine 158 μmol/L), and elevated BNP further heighten risk.

- Moderating factors: Deep hypothermia (23°C) with 31 minutes of lower-body arrest, preserved/reconstructed left subclavian artery, and absence of a bovine arch provide partial protection but do not offset the dominant adverse signals.

- Training data use: Team assessments integrated cohort-level patterns, noting higher risk with femoral cannulation, longer CPB/operative times, lower platelets/lymphocytes, and markedly elevated intraoperative lactate and glucose.

--------------------------------------------------------------------------------

>>>>>>>> TERMINATING RUN: Maximum rounds (17) reached

**Supplementary Data 1**. Complete JSON structure of all candidate variables used for in-context learning.

{

"Training": [

{

"Variable Name": "number",

"Level": null,

"Variable Type": null,

"Representing Method": null,

"Test_Method": null,

"Overall": "533(100%)",

"pnc_no": "464(87.1%)",

"pnc_yes": "69(12.9%)",

"P Value": null,

"Significant": null

},

{

"Variable Name": "age",

"Level": null,

"Variable Type": "continuous",

"Representing Method": "mean value(SD)",

"Test_Method": "Welch t-test",

"Overall": "51.86 (11.96)",

"pnc_no": "51.38 (12.03)",

"pnc_yes": "55.09 (11.06)",

"P Value": 0.0118,

"Significant": "Yes*"

},

{

"Variable Name": "bmi",

"Level": null,

"Variable Type": "continuous ",

"Representing Method": "median(quartile)",

"Test_Method": "Mann-Whitney U Test",

"Overall": "25.18 (22.66, 27.68)",

"pnc_no": "25.22 (22.65, 27.75)",

"pnc_yes": "24.77 (22.86, 27.34)",

"P Value": 0.907,

"Significant": "No"

},

{

"Variable Name": "sex(male)",

"Level": 1.0,

"Variable Type": "categorical",

"Representing Method": "frequency(percentage)",

"Test_Method": "Chi-square Test",

"Overall": "402(75.4%)",

"pnc_no": "353(76.1%)",

"pnc_yes": "49(71.0%)",

"P Value": 0.362,

"Significant": "No"

},

{

"Variable Name": "smoking history",

"Level": 1.0,

"Variable Type": "categorical ",

"Representing Method": "frequency(percentage)",

"Test_Method": "Chi-square Test",

"Overall": "210 (39.4%)",

"pnc_no": "184 (39.7%)",

"pnc_yes": "26 (37.7%)",

"P Value": 0.754,

"Significant": "No"

},

{

"Variable Name": "alcoholic history ",

"Level": 1.0,

"Variable Type": "categorical ",

"Representing Method": "frequency(percentage)",

"Test_Method": "Chi-square Test",

"Overall": "175 (32.8%)",

"pnc_no": "157 (33.8%)",

"pnc_yes": "18 (26.1%)",

"P Value": 0.201,

"Significant": "No"

},

{

"Variable Name": "hypertension",

"Level": 1.0,

"Variable Type": "categorical ",

"Representing Method": "frequency(percentage)",

"Test_Method": "Chi-square Test",

"Overall": "380 (71.3%)",

"pnc_no": "334 (72%)",

"pnc_yes": "46 (66.7%)",

"P Value": 0.362,

"Significant": "No"

},

{

"Variable Name": "arrhythmia",

"Level": 1.0,

"Variable Type": "categorical ",

"Representing Method": "frequency(percentage)",

"Test_Method": "Fisher's Exact Test",

"Overall": "12 (2.3%)",

"pnc_no": "10 (2.2%)",

"pnc_yes": "2 (2.9%)",

"P Value": 0.66,

"Significant": "No"

},

{

"Variable Name": "diabetes",

"Level": 1.0,

"Variable Type": "categorical ",

"Representing Method": "frequency(percentage)",

"Test_Method": "Fisher's Exact Test",

"Overall": "17 (3.2%)",

"pnc_no": "13 (2.8%)",

"pnc_yes": "4 (5.8%)",

"P Value": 0.257,

"Significant": "No"

},

{

"Variable Name": "marfan syndrome",

"Level": 1.0,

"Variable Type": "categorical ",

"Representing Method": "frequency(percentage)",

"Test_Method": "Fisher's Exact Test",

"Overall": "17 (3.2%)",

"pnc_no": "17 (3.7%)",

"pnc_yes": "0 (0%)",

"P Value": 0.147,

"Significant": "No"

},

{

"Variable Name": "renal insufficiency",

"Level": 1.0,

"Variable Type": "categorical ",

"Representing Method": "frequency(percentage)",

"Test_Method": "Chi-square Test",

"Overall": "35 (6.6%)",

"pnc_no": "28 (6%)",

"pnc_yes": "7 (10.1%)",

"P Value": 0.195,

"Significant": "No"

},

{

"Variable Name": "coronary heart disease",

"Level": 1.0,

"Variable Type": "categorical ",

"Representing Method": "frequency(percentage)",

"Test_Method": "Fisher's Exact Test",

"Overall": "11 (2.1%)",

"pnc_no": "8 (1.7%)",

"pnc_yes": "3 (4.3%)",

"P Value": 0.16,

"Significant": "No"

},

{

"Variable Name": "stroke",

"Level": 1.0,

"Variable Type": "categorical ",

"Representing Method": "frequency(percentage)",

"Test_Method": "Chi-square Test",

"Overall": "17 (3.2%)",

"pnc_no": "12 (2.6%)",

"pnc_yes": "5 (7.2%)",

"P Value": 0.0559,

"Significant": "No"

},

{

"Variable Name": "carotid atherosclerosis",

"Level": 1.0,

"Variable Type": "categorical ",

"Representing Method": "frequency(percentage)",

"Test_Method": "Fisher's Exact Test",

"Overall": "2 (0.4%)",

"pnc_no": "2 (0.4%)",

"pnc_yes": "0 (0%)",

"P Value": 1.0,

"Significant": "No"

},

{

"Variable Name": "thrombosis",

"Level": 1.0,

"Variable Type": "categorical ",

"Representing Method": "frequency(percentage)",

"Test_Method": "Fisher's Exact Test",

"Overall": "0 (0.0%)",

"pnc_no": "0 (0.0%)",

"pnc_yes": "0 (0.0%)",

"P Value": 1.0,

"Significant": "No"

},

{

"Variable Name": "valvular heart disease",

"Level": 1.0,

"Variable Type": "categorical ",

"Representing Method": "frequency(percentage)",

"Test_Method": "Chi-square Test",

"Overall": "62 (11.6%)",

"pnc_no": "57 (12.3%)",

"pnc_yes": "5 (7.2%)",

"P Value": 0.223,

"Significant": "No"

},

{

"Variable Name": "chronic obstructive pulmonary disease",

"Level": 1.0,

"Variable Type": "categorical ",

"Representing Method": "frequency(percentage)",

"Test_Method": "Fisher's Exact Test",

"Overall": "23 (4.3%)",

"pnc_no": "21 (4.5%)",

"pnc_yes": "2 (2.9%)",

"P Value": 0.755,

"Significant": "No"

},

{

"Variable Name": "thyroid disease",

"Level": 1.0,

"Variable Type": "categorical ",

"Representing Method": "frequency(percentage)",

"Test_Method": "Fisher's Exact Test",

"Overall": "8 (1.5%)",

"pnc_no": "7 (1.5%)",

"pnc_yes": "1 (1.4%)",

"P Value": 1.0,

"Significant": "No"

},

{

"Variable Name": "presurgical history",

"Level": 1.0,

"Variable Type": "categorical ",

"Representing Method": "frequency(percentage)",

"Test_Method": "Chi-square Test",

"Overall": "79 (14.8%)",

"pnc_no": "69 (14.9%)",

"pnc_yes": "10 (14.5%)",

"P Value": 0.934,

"Significant": "No"

},

{

"Variable Name": "hemoglobin",

"Level": null,

"Variable Type": "continuous ",

"Representing Method": "median(quartile)",

"Test_Method": "Mann-Whitney U Test",

"Overall": "128.00 (111.00, 139.00)",

"pnc_no": "128.00 (112.00, 140.00)",

"pnc_yes": "122.00 (104.00, 136.00)",

"P Value": 0.0532,

"Significant": "No"

},

{

"Variable Name": "wbc",

"Level": null,

"Variable Type": "continuous ",

"Representing Method": "median(quartile)",

"Test_Method": "Mann-Whitney U Test",

"Overall": "10.92 (8.67, 13.73)",

"pnc_no": "10.81 (8.63, 13.66)",

"pnc_yes": "10.97 (8.94, 13.77)",

"P Value": 0.621,

"Significant": "No"

},

{

"Variable Name": "platelet",

"Level": null,

"Variable Type": "continuous ",

"Representing Method": "median(quartile)",

"Test_Method": "Mann-Whitney U Test",

"Overall": "147.00 (106.00, 189.00)",

"pnc_no": "149.00 (108.00, 191.8)",

"pnc_yes": "129.00 (93.50, 170.50)",

"P Value": 0.027,

"Significant": "Yes*"

},

{

"Variable Name": "neutrophil percentage",

"Level": null,

"Variable Type": "continuous ",

"Representing Method": "median(quartile)",

"Test_Method": "Mann-Whitney U Test",

"Overall": "82.80 (77.90, 87.40)",

"pnc_no": "82.70 (77.65, 87.40)",

"pnc_yes": "84.00 (79.35, 88.55)",

"P Value": 0.143,

"Significant": "No"

},

{

"Variable Name": "absolute neutrophil count",

"Level": null,

"Variable Type": "continuous ",

"Representing Method": "median(quartile)",

"Test_Method": "Mann-Whitney U Test",

"Overall": "9.07 (7.00, 11.68)",

"pnc_no": "9.05 (6.99, 11.71)",

"pnc_yes": "9.45 (7.28, 11.41)",

"P Value": 0.555,

"Significant": "No"

},

{

"Variable Name": "lymphocyte",

"Level": null,

"Variable Type": "continuous ",

"Representing Method": "median(quartile)",

"Test_Method": "Mann-Whitney U Test",

"Overall": "0.94 (0.65, 1.29)",

"pnc_no": "0.95 (0.67, 1.3)",

"pnc_yes": "0.87 (0.59, 1.12)",

"P Value": 0.0354,

"Significant": "Yes*"

},

{

"Variable Name": "monocyte",

"Level": null,

"Variable Type": "continuous ",

"Representing Method": "median(quartile)",

"Test_Method": "Mann-Whitney U Test",

"Overall": "0.84 (0.58, 1.14)",

"pnc_no": "0.82 (0.58, 1.13)",

"pnc_yes": "0.87 (0.54, 1.15)",

"P Value": 0.991,

"Significant": "No"

},

{

"Variable Name": "pt",

"Level": null,

"Variable Type": "continuous ",

"Representing Method": "median(quartile)",

"Test_Method": "Mann-Whitney U Test",

"Overall": "11.70 (11.10, 12.50)",

"pnc_no": "11.70 (11.10, 12.50)",

"pnc_yes": "11.80 (11.20, 12.50)",

"P Value": 0.53,

"Significant": "No"

},

{

"Variable Name": "inr",

"Level": null,

"Variable Type": "continuous ",

"Representing Method": "median(quartile)",

"Test_Method": "Mann-Whitney U Test",

"Overall": "1.05 (0.99, 1.12)",

"pnc_no": "1.05 (0.99, 1.11)",

"pnc_yes": "1.07 (1.01, 1.12)",

"P Value": 0.213,

"Significant": "No"

},

{

"Variable Name": "fibrinogen",

"Level": null,

"Variable Type": "continuous ",

"Representing Method": "median(quartile)",

"Test_Method": "Mann-Whitney U Test",

"Overall": "2.80 (2.06, 4.34)",

"pnc_no": "2.84 (2.08, 4.48)",

"pnc_yes": "2.58 (1.99, 3.69)",

"P Value": 0.352,

"Significant": "No"

},

{

"Variable Name": "d-dimer",

"Level": null,

"Variable Type": "continuous ",

"Representing Method": "median(quartile)",

"Test_Method": "Mann-Whitney U Test",

"Overall": "6.36 (2.70, 13.74)",

"pnc_no": "6.15 (2.57, 13.72)",

"pnc_yes": "8.56 (3.88, 16.28)",

"P Value": 0.0828,

"Significant": "No"

},

{

"Variable Name": "tsh",

"Level": null,

"Variable Type": "continuous ",

"Representing Method": "median(quartile)",

"Test_Method": "Mann-Whitney U Test",

"Overall": "1.18 (0.67, 2.06)",

"pnc_no": "1.17 (0.65, 1.98)",

"pnc_yes": "1.36 (0.78, 2.81)",

"P Value": 0.124,

"Significant": "No"

},

{

"Variable Name": "t3",

"Level": null,

"Variable Type": "continuous ",

"Representing Method": "median(quartile)",

"Test_Method": "Mann-Whitney U Test",

"Overall": "1.28 (1.04, 1.53)",

"pnc_no": "1.28 (1.05, 1.53)",

"pnc_yes": "1.29 (1.03, 1.48)",

"P Value": 0.938,

"Significant": "No"

},

{

"Variable Name": "ft3",

"Level": null,

"Variable Type": "continuous ",

"Representing Method": "median(quartile)",

"Test_Method": "Mann-Whitney U Test",

"Overall": "3.69 (3.07, 4.22)",

"pnc_no": "3.72 (3.10, 4.22)",

"pnc_yes": "3.48 (2.92, 4.17)",

"P Value": 0.138,

"Significant": "No"

},

{

"Variable Name": "t4",

"Level": null,

"Variable Type": "continuous ",

"Representing Method": "median(quartile)",

"Test_Method": "Mann-Whitney U Test",

"Overall": "81.50 (71, 95.1)",

"pnc_no": "81.55 (70.90, 95.12)",

"pnc_yes": "80.30 (71.60, 94.01)",

"P Value": 0.976,

"Significant": "No"

},

{

"Variable Name": "ft4",

"Level": null,

"Variable Type": "continuous ",

"Representing Method": "median(quartile)",

"Test_Method": "Mann-Whitney U Test",

"Overall": "15.10 (13.40, 16.60)",

"pnc_no": "15.10(13.50, 16.60)",

"pnc_yes": "15.10 (13.05, 16.90)",

"P Value": 0.972,

"Significant": "No"

},

{

"Variable Name": "bilirubin",

"Level": null,

"Variable Type": "continuous ",

"Representing Method": "median(quartile)",

"Test_Method": "Mann-Whitney U Test",

"Overall": "15.90 (11.10, 22.50)",

"pnc_no": "15.95 (11.10, 22.52)",

"pnc_yes": "15.60 (11.00, 20.70)",

"P Value": 0.768,

"Significant": "No"

},

{

"Variable Name": "hdl",

"Level": null,

"Variable Type": "continuous ",

"Representing Method": "median(quartile)",

"Test_Method": "Mann-Whitney U Test",

"Overall": "1.11 (0.91, 1.35)",

"pnc_no": "1.11 (0.91, 1.34)",

"pnc_yes": "1.14 (0.96, 1.46)",

"P Value": 0.249,

"Significant": "No"

},

{

"Variable Name": "ldl",

"Level": null,

"Variable Type": "continuous ",

"Representing Method": "median(quartile)",

"Test_Method": "Mann-Whitney U Test",

"Overall": "2.23 (1.68, 2.8)",

"pnc_no": "2.21 (1.68, 2.82)",

"pnc_yes": "2.34 (1.75, 2.74)",

"P Value": 0.985,

"Significant": "No"

},

{

"Variable Name": "triglycerides",

"Level": null,

"Variable Type": "continuous ",

"Representing Method": "median(quartile)",

"Test_Method": "Mann-Whitney U Test",

"Overall": "1.17 (0.84, 1.64)",

"pnc_no": "1.18 (0.83, 1.64)",

"pnc_yes": "1.17 (0.91, 1.51)",

"P Value": 0.859,

"Significant": "No"

},

{

"Variable Name": "total cholesterol",

"Level": null,

"Variable Type": "continuous ",

"Representing Method": "median(quartile)",

"Test_Method": "Mann-Whitney U Test",

"Overall": "3.83 (3.21, 4.47)",

"pnc_no": "3.83 (3.21, 4.49)",

"pnc_yes": "3.99 (3.25, 4.36)",

"P Value": 0.884,

"Significant": "No"

},

{

"Variable Name": "uric acid",

"Level": null,

"Variable Type": "continuous ",

"Representing Method": "median(quartile)",

"Test_Method": "Mann-Whitney U Test",

"Overall": "360.00 (282.00, 451.00)",

"pnc_no": "359.00 (281.25, 444.25)",

"pnc_yes": "369.00 (293.00, 480.00)",

"P Value": 0.404,

"Significant": "No"

},

{

"Variable Name": "creatinine",

"Level": null,

"Variable Type": "continuous ",

"Representing Method": "median(quartile)",

"Test_Method": "Mann-Whitney U Test",

"Overall": "90.00 (72.00, 117.00)",

"pnc_no": "89.50 (70.00, 114.00)",

"pnc_yes": "100.00 (81.00, 138.00)",

"P Value": 0.0653,

"Significant": "No"

},

{

"Variable Name": "preoperative fasting blood glucose",

"Level": null,

"Variable Type": "continuous ",

"Representing Method": "median(quartile)",

"Test_Method": "Mann-Whitney U Test",

"Overall": "6.90 (5.92, 8.14)",

"pnc_no": "6.82 (5.83, 7.96)",

"pnc_yes": "7.32 (6.49, 8.74)",

"P Value": 0.00428,

"Significant": "Yes*"

},

{

"Variable Name": "albumin",

"Level": null,

"Variable Type": "continuous ",

"Representing Method": "median(quartile)",

"Test_Method": "Mann-Whitney U Test",

"Overall": "39.10 (36.40, 41.70)",

"pnc_no": "39.20 (36.40, 41.80)",

"pnc_yes": "38.40 (36.20, 41.50)",

"P Value": 0.314,

"Significant": "No"

},

{

"Variable Name": "alt",

"Level": null,

"Variable Type": "continuous ",

"Representing Method": "median(quartile)",

"Test_Method": "Mann-Whitney U Test",

"Overall": "21.00 (14.00, 38.00)",

"pnc_no": "22.00 (14.00, 39.00)",

"pnc_yes": "18.00 (14.00, 33.00)",

"P Value": 0.256,

"Significant": "No"

},

{

"Variable Name": "ast",

"Level": null,

"Variable Type": "continuous ",

"Representing Method": "median(quartile)",

"Test_Method": "Mann-Whitney U Test",

"Overall": "22.00 (16.00, 40.00)",

"pnc_no": "22.00 (16.00, 39.00)",

"pnc_yes": "23.00 (15.00, 47.00)",

"P Value": 0.788,

"Significant": "No"

},

{

"Variable Name": "preoperative myoglobin",

"Level": null,

"Variable Type": "continuous ",

"Representing Method": "median(quartile)",

"Test_Method": "Mann-Whitney U Test",

"Overall": "41.83 (24.01, 100.80)",

"pnc_no": "40.11 (23.85, 91.05)",

"pnc_yes": "83.25 (27.59, 221.30)",

"P Value": 0.00263,

"Significant": "Yes*"

},

{

"Variable Name": "bnp",

"Level": null,

"Variable Type": "continuous ",

"Representing Method": "median(quartile)",

"Test_Method": "Mann-Whitney U Test",

"Overall": "429.00 (173.00, 990.00)",

"pnc_no": "421.00 (166.50, 967.50)",

"pnc_yes": "514.00 (246.00, 1543.00)",

"P Value": 0.0521,

"Significant": "No"

},

{

"Variable Name": "crp",

"Level": null,

"Variable Type": "continuous ",

"Representing Method": "median(quartile)",

"Test_Method": "Mann-Whitney U Test",

"Overall": "58.00 (13.50, 117.00)",

"pnc_no": "64.05 (14.35, 118.25)",

"pnc_yes": "34.20 (11.90, 106.00)",

"P Value": 0.278,

"Significant": "No"

},

{

"Variable Name": "preoperative PaO2",

"Level": null,

"Variable Type": "continuous ",

"Representing Method": "median(quartile)",

"Test_Method": "Mann-Whitney U Test",

"Overall": "95.30 (76.40, 123.20)",

"pnc_no": "94.50 (76.57, 121.20)",

"pnc_yes": "99.60 (74.50, 129.10)",

"P Value": 0.522,

"Significant": "No"

},

{

"Variable Name": "preoperative PaCO2",

"Level": null,

"Variable Type": "continuous ",

"Representing Method": "mean value(SD)",

"Test_Method": "Welch t-test",

"Overall": "36.12 (6.45)",

"pnc_no": "35.98 (6.18)",

"pnc_yes": "37.06 (8.02)",

"P Value": 0.283,

"Significant": "No"

},

{

"Variable Name": "preoperative hypotension",

"Level": 1.0,

"Variable Type": "categorical ",

"Representing Method": "frequency(percentage)",

"Test_Method": "Chi-square Test",

"Overall": "74 (13.9%)",

"pnc_no": "61 (13.1%)",

"pnc_yes": "13 (18.8%)",

"P Value": 0.202,

"Significant": "No"

},

{

"Variable Name": "preoperative alteration of consciousness state",

"Level": 1.0,

"Variable Type": "categorical ",

"Representing Method": "frequency(percentage)",

"Test_Method": "Chi-square Test",

"Overall": "61 (11.4%)",

"pnc_no": "50 (10.8%)",

"pnc_yes": "11 (15.9%)",

"P Value": 0.208,

"Significant": "No"

},

{

"Variable Name": "preoperative limb ischemia",

"Level": 1.0,

"Variable Type": "categorical ",

"Representing Method": "frequency(percentage)",

"Test_Method": "Chi-square Test",

"Overall": "101 (18.9%)",

"pnc_no": "81 (17.5%)",

"pnc_yes": "20 (29%)",

"P Value": 0.0226,

"Significant": "Yes*"

},

{

"Variable Name": "entry tear=unknown",

"Level": 0.0,

"Variable Type": "categorical ",

"Representing Method": "frequency(percentage)",

"Test_Method": "Fisher's Exact Test",

"Overall": "13 (2.4%)",

"pnc_no": "12 (2.6%)",

"pnc_yes": "1 (1.4%)",

"P Value": 0.162,

"Significant": "No"

},

{

"Variable Name": "entry tear=ascending aorta",

"Level": 1.0,

"Variable Type": "categorical ",

"Representing Method": "frequency(percentage)",

"Test_Method": "Fisher's Exact Test",

"Overall": "387 (72.6%)",

"pnc_no": "329 (70.9%)",

"pnc_yes": "58 (84.1%)",

"P Value": 0.162,

"Significant": "No"

},

{

"Variable Name": "entry tear=arcus aortae",

"Level": 2.0,

"Variable Type": "categorical ",

"Representing Method": "frequency(percentage)",

"Test_Method": "Fisher's Exact Test",

"Overall": "109 (20.5%)",

"pnc_no": "101 (21.8%)",

"pnc_yes": "8 (11.6%)",

"P Value": 0.162,

"Significant": "No"

},

{

"Variable Name": "entry tear=descending aorta",

"Level": 3.0,

"Variable Type": "categorical ",

"Representing Method": "frequency(percentage)",

"Test_Method": "Fisher's Exact Test",

"Overall": "24 (4.5%)",

"pnc_no": "22 (4.7%)",

"pnc_yes": "2 (2.9%)",

"P Value": 0.162,

"Significant": "No"

},

{

"Variable Name": "retrograde dissection",

"Level": 1.0,

"Variable Type": "categorical",

"Representing Method": "frequency(percentage)",

"Test_Method": "Fisher's Exact Test",

"Overall": "15 (2.8%)",

"pnc_no": "13 (2.8%)",

"pnc_yes": "2 (2.9%)",

"P Value": 1.0,

"Significant": "No"

},

{

"Variable Name": "sma originating from the true cavity",

"Level": 0.0,

"Variable Type": "categorical ",

"Representing Method": "frequency(percentage)",

"Test_Method": "Fisher's Exact Test",

"Overall": "385 (72.2%)",

"pnc_no": "336 (72.4%)",

"pnc_yes": "49 (71%)",

"P Value": 0.942,

"Significant": "No"

},

{

"Variable Name": "partial involvement of the sma",

"Level": 1.0,

"Variable Type": "categorical ",

"Representing Method": "frequency(percentage)",

"Test_Method": "Fisher's Exact Test",

"Overall": "122 (22.9%)",

"pnc_no": "105 (22.6%)",

"pnc_yes": "17 (24.6%)",

"P Value": 0.942,

"Significant": "No"

},

{

"Variable Name": "severe involvement of the sma",

"Level": 2.0,

"Variable Type": "categorical ",

"Representing Method": "frequency(percentage)",

"Test_Method": "Fisher's Exact Test",

"Overall": "26 (4.9%)",

"pnc_no": "23 (5%)",

"pnc_yes": "3 (4.3%)",

"P Value": 0.942,

"Significant": "No"

},

{

"Variable Name": "bovine arch",

"Level": 1.0,

"Variable Type": "categorical ",

"Representing Method": "frequency(percentage)",

"Test_Method": "Fisher's Exact Test",

"Overall": "10 (1.9%)",

"pnc_no": "6 (1.3%)",

"pnc_yes": "4 (5.8%)",

"P Value": 0.0295,

"Significant": "Yes*"

},

{

"Variable Name": "pericardial effusion volume=normal",

"Level": 0.0,

"Variable Type": "categorical ",

"Representing Method": "frequency(percentage)",

"Test_Method": "Fisher's Exact Test",

"Overall": "42 (7.9%)",

"pnc_no": "37 (8%)",

"pnc_yes": "5 (7.2%)",

"P Value": 1.0,

"Significant": "No"

},

{

"Variable Name": "pericardial effusion volume=few",

"Level": 1.0,

"Variable Type": "categorical ",

"Representing Method": "frequency(percentage)",

"Test_Method": "Fisher's Exact Test",

"Overall": "377 (70.7%)",

"pnc_no": "327 (70.5%)",

"pnc_yes": "50 (72.5%)",

"P Value": 1.0,

"Significant": "No"

},

{

"Variable Name": "pericardial effusion volume=medium",

"Level": 2.0,

"Variable Type": "categorical ",

"Representing Method": "frequency(percentage)",

"Test_Method": "Fisher's Exact Test",

"Overall": "88 (16.5%)",

"pnc_no": "77 (16.6%)",

"pnc_yes": "11 (15.9%)",

"P Value": 1.0,

"Significant": "No"

},

{

"Variable Name": "pericardial effusion volume=large",

"Level": 3.0,

"Variable Type": "categorical ",

"Representing Method": "frequency(percentage)",

"Test_Method": "Fisher's Exact Test",

"Overall": "26 (4.9%)",

"pnc_no": "23 (5%)",

"pnc_yes": "3 (4.3%)",

"P Value": 1.0,

"Significant": "No"

},

{

"Variable Name": "arch replacement=no",

"Level": 0.0,

"Variable Type": "categorical ",

"Representing Method": "frequency(percentage)",

"Test_Method": "Chi-square Test",

"Overall": "40 (7.5%)",

"pnc_no": "34 (7.3%)",

"pnc_yes": "6 (8.7%)",

"P Value": 0.278,

"Significant": "No"

},

{

"Variable Name": "arch replacement=hemi arch replacement",

"Level": 1.0,

"Variable Type": "categorical ",

"Representing Method": "frequency(percentage)",

"Test_Method": "Chi-square Test",

"Overall": "104 (19.5%)",

"pnc_no": "86 (18.5%)",

"pnc_yes": "18 (26.1%)",

"P Value": 0.278,

"Significant": "No"

},

{

"Variable Name": "arch replacement=total arch replacement",

"Level": 2.0,

"Variable Type": "categorical ",

"Representing Method": "frequency(percentage)",

"Test_Method": "Chi-square Test",

"Overall": "389 (73%)",

"pnc_no": "344 (74.1%)",

"pnc_yes": "45 (65.2%)",

"P Value": 0.278,

"Significant": "No"

},

{

"Variable Name": "valve replacement=no",

"Level": 0.0,

"Variable Type": "categorical ",

"Representing Method": "frequency(percentage)",

"Test_Method": "Chi-square Test",

"Overall": "66 (12.4%)",

"pnc_no": "58 (12.5%)",

"pnc_yes": "8 (11.6%)",

"P Value": 0.957,

"Significant": "No"

},

{

"Variable Name": "valve replacement=shaping",

"Level": 1.0,

"Variable Type": "categorical ",

"Representing Method": "frequency(percentage)",

"Test_Method": "Chi-square Test",

"Overall": "270 (50.7%)",

"pnc_no": "234 (50.4%)",

"pnc_yes": "36 (52.2%)",

"P Value": 0.957,

"Significant": "No"

},

{

"Variable Name": "valve replacement=yes",

"Level": 2.0,

"Variable Type": "categorical ",

"Representing Method": "frequency(percentage)",

"Test_Method": "Chi-square Test",

"Overall": "197 (37%)",

"pnc_no": "172 (37.1%)",

"pnc_yes": "25 (36.2%)",

"P Value": 0.957,

"Significant": "No"

},

{

"Variable Name": "coronary artery bypass grafting",

"Level": 1.0,

"Variable Type": "categorical ",

"Representing Method": "frequency(percentage)",

"Test_Method": "Chi-square Test",

"Overall": "47 (8.8%)",

"pnc_no": "39 (8.4%)",

"pnc_yes": "8 (11.6%)",

"P Value": 0.383,

"Significant": "No"

},

{

"Variable Name": "ligation of sca without subsequent reconstruction",

"Level": 1.0,

"Variable Type": "categorical ",

"Representing Method": "frequency(percentage)",

"Test_Method": "Chi-square Test",

"Overall": "29 (5.4%)",

"pnc_no": "21 (4.5%)",

"pnc_yes": "8 (11.6%)",

"P Value": 0.0397,

"Significant": "Yes*"

},

{

"Variable Name": "operation duration",

"Level": null,

"Variable Type": "continuous ",

"Representing Method": "median(quartile)",

"Test_Method": "Mann-Whitney U Test",

"Overall": "6.90 (6.10, 8.00)",

"pnc_no": "6.80 (6.00, 8.00)",

"pnc_yes": "7.48 (6.50, 8.30)",

"P Value": 0.00529,

"Significant": "Yes*"

},

{

"Variable Name": "lowest temperature",

"Level": null,

"Variable Type": "continuous ",

"Representing Method": "median(quartile)",

"Test_Method": "Mann-Whitney U Test",

"Overall": "24.00 (23.30, 24.70)",

"pnc_no": "24.00 (23.30, 24.70)",

"pnc_yes": "23.90 (23.30, 24.80)",

"P Value": 0.422,

"Significant": "No"

},

{

"Variable Name": "cpb time",

"Level": null,

"Variable Type": "continuous ",

"Representing Method": "median(quartile)",

"Test_Method": "Mann-Whitney U Test",

"Overall": "192.00 (164.00, 231.00)",

"pnc_no": "190.00 (162.00, 226.00)",

"pnc_yes": "208.00 (176.00, 259.00)",

"P Value": 0.0131,

"Significant": "Yes*"

},

{

"Variable Name": "acc time",

"Level": null,

"Variable Type": "continuous ",

"Representing Method": "median(quartile)",

"Test_Method": "Mann-Whitney U Test",

"Overall": "141.00 (112.00, 175.00)",

"pnc_no": "138.50 (111.00, 172.00)",

"pnc_yes": "156.00 (114.00, 193.00)",

"P Value": 0.0841,

"Significant": "No"

},

{

"Variable Name": "lower extremity occlusion time",

"Level": null,

"Variable Type": "continuous ",

"Representing Method": "median(quartile)",

"Test_Method": "Mann-Whitney U Test",

"Overall": "25.00 (19.00, 31.00)",

"pnc_no": "25.00 (18.00, 30.00)",

"pnc_yes": "26.00 (20.00, 36.00)",

"P Value": 0.141,

"Significant": "No"

},

{

"Variable Name": "cannulation site=axillary artery",

"Level": 1.0,

"Variable Type": "categorical ",

"Representing Method": "frequency(percentage)",

"Test_Method": "Fisher's Exact Test",

"Overall": "94 (17.6%)",

"pnc_no": "87 (18.8%)",

"pnc_yes": "7 (10.1%)",

"P Value": 0.0273,

"Significant": "Yes*"

},

{

"Variable Name": "cannulation site=arteria cruralis",

"Level": 2.0,

"Variable Type": "categorical ",

"Representing Method": "frequency(percentage)",

"Test_Method": "Fisher's Exact Test",

"Overall": "406 (76.2%)",

"pnc_no": "351 (75.6%)",

"pnc_yes": "55 (79.7%)",

"P Value": 0.0273,

"Significant": "Yes*"

},

{

"Variable Name": "cannulation site=axillary artery+arteria cruralis",

"Level": 3.0,

"Variable Type": "categorical ",

"Representing Method": "frequency(percentage)",

"Test_Method": "Fisher's Exact Test",

"Overall": "15 (2.8%)",

"pnc_no": "14 (3%)",

"pnc_yes": "1 (1.4%)",

"P Value": 0.0273,

"Significant": "Yes*"

},

{

"Variable Name": "cannulation site=other",

"Level": 4.0,

"Variable Type": "categorical ",

"Representing Method": "frequency(percentage)",

"Test_Method": "Fisher's Exact Test",

"Overall": "18 (3.4%)",

"pnc_no": "12 (2.6%)",

"pnc_yes": "6 (8.7%)",

"P Value": 0.0273,

"Significant": "Yes*"

},

{

"Variable Name": "cerebral perfusion=no",

"Level": 0.0,

"Variable Type": "categorical ",

"Representing Method": "frequency(percentage)",

"Test_Method": "Chi-square Test",

"Overall": "68 (12.8%)",

"pnc_no": "57 (12.3%)",

"pnc_yes": "11 (15.9%)",

"P Value": 0.13,

"Significant": "No"

},

{

"Variable Name": "cerebral perfusion=sequential perfusion,unilateral",

"Level": 1.0,

"Variable Type": "categorical ",

"Representing Method": "frequency(percentage)",

"Test_Method": "Chi-square Test",

"Overall": "337 (63.2%)",

"pnc_no": "297 (64%)",

"pnc_yes": "40 (58%)",

"P Value": 0.13,

"Significant": "No"

},

{

"Variable Name": "cerebral perfusion=sequential perfusion,bilateral",

"Level": 2.0,

"Variable Type": "categorical ",

"Representing Method": "frequency(percentage)",

"Test_Method": "Chi-square Test",

"Overall": "57 (10.7%)",

"pnc_no": "53 (11.4%)",

"pnc_yes": "4 (5.8%)",

"P Value": 0.13,

"Significant": "No"

},

{

"Variable Name": "cerebral perfusion=retrograde perfusion",

"Level": 3.0,

"Variable Type": "categorical ",

"Representing Method": "frequency(percentage)",

"Test_Method": "Chi-square Test",

"Overall": "71 (13.3%)",

"pnc_no": "57 (12.3%)",

"pnc_yes": "14 (20.3%)",

"P Value": 0.13,

"Significant": "No"

},

{

"Variable Name": "intraoperative plasma transfusion",

"Level": null,

"Variable Type": "continuous ",

"Representing Method": "median(quartile)",

"Test_Method": "Mann-Whitney U Test",

"Overall": "0.00 (0.00, 200.0)",

"pnc_no": "0.00 (0.00, 0.00)",

"pnc_yes": "0.00 (0.00, 400.00)",

"P Value": 0.101,

"Significant": "No"

},

{

"Variable Name": "intraoperative packed red blood cell transfusion",

"Level": null,

"Variable Type": "continuous",

"Representing Method": "median(quartile)",

"Test_Method": "Mann-Whitney U Test",

"Overall": "0.00 (0.00, 2.00)",

"pnc_no": "0.00 (0.00, 2.00)",

"pnc_yes": "0.00 (0.00, 2.00)",

"P Value": 0.128,

"Significant": "No"

},

{

"Variable Name": "intraoperative platelets transfusion",

"Level": null,

"Variable Type": "continuous",

"Representing Method": "median(quartile)",

"Test_Method": "Mann-Whitney U Test",

"Overall": "2.00(1.00,2.00)",

"pnc_no": "1.00(1.00,2.00)",

"pnc_yes": "2.00(1.00,2.00)",

"P Value": 0.000164,

"Significant": "Yes*"

},

{

"Variable Name": "fluid volume",

"Level": null,

"Variable Type": "continuous ",

"Representing Method": "median(quartile)",

"Test_Method": "Mann-Whitney U Test",

"Overall": "1600.00 (1300.00, 2100.00)",

"pnc_no": "1600.00 (1200.00, 2100.00)",

"pnc_yes": "1600.00 (1500.00, 2300.00)",

"P Value": 0.207,

"Significant": "No"

},

{

"Variable Name": "max glucose intraoperation",

"Level": null,

"Variable Type": "continuous ",

"Representing Method": "median(quartile)",

"Test_Method": "Mann-Whitney U Test",

"Overall": "10.20 (8.50, 12.20)",

"pnc_no": "10.05 (8.50, 12.10)",

"pnc_yes": "11.20 (8.50, 13.50)",

"P Value": 0.089,

"Significant": "No"

},

{

"Variable Name": "max lactate intraoperation",

"Level": null,

"Variable Type": "continuous ",

"Representing Method": "median(quartile)",

"Test_Method": "Mann-Whitney U Test",

"Overall": "5.20 (3.90, 8.20)",

"pnc_no": "4.95 (3.70, 7.53)",

"pnc_yes": "7.30 (4.90, 10.70)",

"P Value": 0.000159,

"Significant": "Yes*"

}

]

}

**Supplementary Data 2**. Complete JSON structure of the 15 LASSO-selected predictors used for in-context learning.

{

"Training": [

{

"Variable Name": "number",

"Level": null,

"Variable Type": null,

"Representing Method": null,

"Test_Method": null,

"Overall": "533(100%)",

"pnc_no": "464(87.1%)",

"pnc_yes": "69(12.9%)",

"P Value": null,

"Significant": null

},

{

"Variable Name": "age",

"Level": null,

"Variable Type": "continuous",

"Representing Method": "mean value(SD)",

"Test_Method": "Welch t-test",

"Overall": "51.86 (11.96)",

"pnc_no": "51.38 (12.03)",

"pnc_yes": "55.09 (11.06)",

"P Value": 0.0118,

"Significant": "Yes*"

},

{

"Variable Name": "stroke",

"Level": 1.0,

"Variable Type": "categorical ",

"Representing Method": "frequency(percentage)",

"Test_Method": "Chi-square Test",

"Overall": "17 (3.2%)",

"pnc_no": "12 (2.6%)",

"pnc_yes": "5 (7.2%)",

"P Value": 0.0559,

"Significant": "No"

},

{

"Variable Name": "hemoglobin",

"Level": null,

"Variable Type": "continuous ",

"Representing Method": "median(quartile)",

"Test_Method": "Mann-Whitney U Test",

"Overall": "128.00 (111.00, 139.00)",

"pnc_no": "128.00 (112.00, 140.00)",

"pnc_yes": "122.00 (104.00, 136.00)",

"P Value": 0.0532,

"Significant": "No"

},

{

"Variable Name": "hdl",

"Level": null,

"Variable Type": "continuous ",

"Representing Method": "median(quartile)",

"Test_Method": "Mann-Whitney U Test",

"Overall": "1.11 (0.91, 1.35)",

"pnc_no": "1.11 (0.91, 1.34)",

"pnc_yes": "1.14 (0.96, 1.46)",

"P Value": 0.249,

"Significant": "No"

},

{

"Variable Name": "preoperative fasting blood glucose",

"Level": null,

"Variable Type": "continuous ",

"Representing Method": "median(quartile)",

"Test_Method": "Mann-Whitney U Test",

"Overall": "6.90 (5.92, 8.14)",

"pnc_no": "6.82 (5.83, 7.96)",

"pnc_yes": "7.32 (6.49, 8.74)",

"P Value": 0.00428,

"Significant": "Yes*"

},

{

"Variable Name": "preoperative myoglobin",

"Level": null,

"Variable Type": "continuous ",

"Representing Method": "median(quartile)",

"Test_Method": "Mann-Whitney U Test",

"Overall": "41.83 (24.01, 100.80)",

"pnc_no": "40.11 (23.85, 91.05)",

"pnc_yes": "83.25 (27.59, 221.30)",

"P Value": 0.00263,

"Significant": "Yes*"

},

{

"Variable Name": "preoperative limb ischemia",

"Level": 1.0,

"Variable Type": "categorical ",

"Representing Method": "frequency(percentage)",

"Test_Method": "Chi-square Test",

"Overall": "101 (18.9%)",

"pnc_no": "81 (17.5%)",

"pnc_yes": "20 (29%)",

"P Value": 0.0226,

"Significant": "Yes*"

},

{

"Variable Name": "entry tear=unknown",

"Level": 0.0,

"Variable Type": "categorical ",

"Representing Method": "frequency(percentage)",

"Test_Method": "Fisher's Exact Test",

"Overall": "13 (2.4%)",

"pnc_no": "12 (2.6%)",

"pnc_yes": "1 (1.4%)",

"P Value": 0.162,

"Significant": "No"

},

{

"Variable Name": "entry tear=ascending aorta",

"Level": 1.0,

"Variable Type": "categorical ",

"Representing Method": "frequency(percentage)",

"Test_Method": "Fisher's Exact Test",

"Overall": "387 (72.6%)",

"pnc_no": "329 (70.9%)",

"pnc_yes": "58 (84.1%)",

"P Value": 0.162,

"Significant": "No"

},

{

"Variable Name": "entry tear=arcus aortae",

"Level": 2.0,

"Variable Type": "categorical ",

"Representing Method": "frequency(percentage)",

"Test_Method": "Fisher's Exact Test",

"Overall": "109 (20.5%)",

"pnc_no": "101 (21.8%)",

"pnc_yes": "8 (11.6%)",

"P Value": 0.162,

"Significant": "No"

},

{

"Variable Name": "entry tear=descending aorta",

"Level": 3.0,

"Variable Type": "categorical ",

"Representing Method": "frequency(percentage)",

"Test_Method": "Fisher's Exact Test",

"Overall": "24 (4.5%)",

"pnc_no": "22 (4.7%)",

"pnc_yes": "2 (2.9%)",

"P Value": 0.162,

"Significant": "No"

},

{

"Variable Name": "bovine arch",

"Level": 1.0,

"Variable Type": "categorical ",

"Representing Method": "frequency(percentage)",

"Test_Method": "Fisher's Exact Test",

"Overall": "10 (1.9%)",

"pnc_no": "6 (1.3%)",

"pnc_yes": "4 (5.8%)",

"P Value": 0.0295,

"Significant": "Yes*"

},

{

"Variable Name": "ligation of sca without subsequent reconstruction",

"Level": 1.0,

"Variable Type": "categorical ",

"Representing Method": "frequency(percentage)",

"Test_Method": "Chi-square Test",

"Overall": "29 (5.4%)",

"pnc_no": "21 (4.5%)",

"pnc_yes": "8 (11.6%)",

"P Value": 0.0397,

"Significant": "Yes*"

},

{

"Variable Name": "cpb time",

"Level": null,

"Variable Type": "continuous ",

"Representing Method": "median(quartile)",

"Test_Method": "Mann-Whitney U Test",

"Overall": "192.00 (164.00, 231.00)",

"pnc_no": "190.00 (162.00, 226.00)",

"pnc_yes": "208.00 (176.00, 259.00)",

"P Value": 0.0131,

"Significant": "Yes*"

},

{

"Variable Name": "cannulation site=axillary artery",

"Level": 1.0,

"Variable Type": "categorical ",

"Representing Method": "frequency(percentage)",

"Test_Method": "Fisher's Exact Test",

"Overall": "94 (17.6%)",

"pnc_no": "87 (18.8%)",

"pnc_yes": "7 (10.1%)",

"P Value": 0.0273,

"Significant": "Yes*"

},

{

"Variable Name": "cannulation site=arteria cruralis",

"Level": 2.0,

"Variable Type": "categorical ",

"Representing Method": "frequency(percentage)",

"Test_Method": "Fisher's Exact Test",

"Overall": "406 (76.2%)",

"pnc_no": "351 (75.6%)",

"pnc_yes": "55 (79.7%)",

"P Value": 0.0273,

"Significant": "Yes*"

},

{

"Variable Name": "cannulation site=axillary artery+arteria cruralis",

"Level": 3.0,

"Variable Type": "categorical ",

"Representing Method": "frequency(percentage)",

"Test_Method": "Fisher's Exact Test",

"Overall": "15 (2.8%)",

"pnc_no": "14 (3%)",

"pnc_yes": "1 (1.4%)",

"P Value": 0.0273,

"Significant": "Yes*"

},

{

"Variable Name": "cannulation site=other",

"Level": 4.0,

"Variable Type": "categorical ",

"Representing Method": "frequency(percentage)",

"Test_Method": "Fisher's Exact Test",

"Overall": "18 (3.4%)",

"pnc_no": "12 (2.6%)",

"pnc_yes": "6 (8.7%)",

"P Value": 0.0273,

"Significant": "Yes*"

},

{

"Variable Name": "cerebral perfusion=no",

"Level": 0.0,

"Variable Type": "categorical ",

"Representing Method": "frequency(percentage)",

"Test_Method": "Chi-square Test",

"Overall": "68 (12.8%)",

"pnc_no": "57 (12.3%)",

"pnc_yes": "11 (15.9%)",

"P Value": 0.13,

"Significant": "No"

},

{

"Variable Name": "cerebral perfusion=sequential perfusion,unilateral",

"Level": 1.0,

"Variable Type": "categorical ",

"Representing Method": "frequency(percentage)",

"Test_Method": "Chi-square Test",

"Overall": "337 (63.2%)",

"pnc_no": "297 (64%)",

"pnc_yes": "40 (58%)",

"P Value": 0.13,

"Significant": "No"

},

{

"Variable Name": "cerebral perfusion=sequential perfusion,bilateral",

"Level": 2.0,

"Variable Type": "categorical ",

"Representing Method": "frequency(percentage)",

"Test_Method": "Chi-square Test",

"Overall": "57 (10.7%)",

"pnc_no": "53 (11.4%)",

"pnc_yes": "4 (5.8%)",

"P Value": 0.13,

"Significant": "No"

},

{

"Variable Name": "cerebral perfusion=retrograde perfusion",

"Level": 3.0,

"Variable Type": "categorical ",

"Representing Method": "frequency(percentage)",

"Test_Method": "Chi-square Test",

"Overall": "71 (13.3%)",

"pnc_no": "57 (12.3%)",

"pnc_yes": "14 (20.3%)",

"P Value": 0.13,

"Significant": "No"

},

{

"Variable Name": "intraoperative platelets transfusion",

"Level": null,

"Variable Type": "continuous",

"Representing Method": "median(quartile)",

"Test_Method": "Mann-Whitney U Test",

"Overall": "2.00(1.00,2.00)",

"pnc_no": "1.00(1.00,2.00)",

"pnc_yes": "2.00(1.00,2.00)",

"P Value": 0.000164,

"Significant": "Yes*"

},

{

"Variable Name": "max lactate intraoperation",

"Level": null,

"Variable Type": "continuous ",

"Representing Method": "median(quartile)",

"Test_Method": "Mann-Whitney U Test",

"Overall": "5.20 (3.90, 8.20)",

"pnc_no": "4.95 (3.70, 7.53)",

"pnc_yes": "7.30 (4.90, 10.70)",

"P Value": 0.000159,

"Significant": "Yes*"

}

]

}
